# Machine Learning Strategies for the Retrieval of Leaf-Chlorophyll Dynamics: Model Choice, Sequential Versus Retraining Learning, and Hyperspectral Predictors

**DOI:** 10.3389/fpls.2022.722442

**Published:** 2022-03-11

**Authors:** Yoseline Angel, Matthew F. McCabe

**Affiliations:** Hydrology, Agriculture and Land Observation Group, Water Desalination and Reuse Center, King Abdullah University of Science and Technology, Thuwal, Saudi Arabia

**Keywords:** chlorophyll, hyperspectral image, SPAD – leaf greenness, machine learning, UAV, multitemporal analyses, vegetation indices, digital phenotyping

## Abstract

Monitoring leaf Chlorophyll (Chl) *in-situ* is labor-intensive, limiting representative sampling for detailed mapping of Chl variability at field scales across time. Unmanned aeria-l vehicles (UAV) and hyperspectral cameras provide flexible platforms for observing agricultural systems, overcoming this spatio-temporal sampling constraint. Here, we evaluate a customized machine learning (ML) workflow to retrieve multi-temporal leaf-Chl levels, combining sub-centimeter resolution UAV-hyperspectral imagery (400–1,000 nm) with leaf-level reflectance spectra and SPAD measurements, capturing temporal correlations, selecting relevant predictors, and retrieving accurate results under different conditions. The study is performed within a phenotyping experiment to monitor wild tomato plants’ development. Several analyses were conducted to evaluate multiple ML strategies, including: (1) exploring sequential versus retraining learning; (2) comparing insights gained from using 272 spectral bands versus 60 pigment-based vegetation indices (VIs); and (3) assessing six regression methods (linear, partial-least-square regression; PLSR, decision trees, support vector, ensemble trees, and Gaussian process; GPR). Goodness-of-fit (*R*^2^) and accuracy metrics (MAE, RMSE) were determined using training/testing and validation data subsets to assess the models’ performance. Overall, while equally good performance was obtained using either PLSR, GPR, or random forest, results show: (1) the retraining strategy improved the ability of most of the approaches to model SPAD-based Chl dynamics; (2) comparative analysis between retrievals and validation data distributions informed the models’ ability to capture Chl dynamics through SPAD levels; (3) VI predictors slightly improved *R*^2^ (e.g., from 0.59 to 0.74 units for GPR) and accuracy (e.g., MAE and RMSE differences of up to 2 SPAD units) in specific algorithms; (4) feature importance examined through these methods, revealed strong overlaps between relevant bands and VI predictors, highlighting a few decisive spectral ranges and indices useful for retrieving leaf-Chl levels. The proposed ML framework allows the retrieval of high-quality spatially distributed and multi-temporal SPAD-based chlorophyll maps at an ultra-high pixel resolution (e.g., 7 mm).

## Introduction

Chlorophyll (Chl) is the primary pigment that drives the exchange of energy required for sugar production through photosynthesis, which ultimately sustains life, produces oxygen, and regulates CO_2_ for the entire planet. From the interaction of visible solar radiation with leaves (approximately 400–750 nm), around 85% is absorbed by leaf pigments to fuel the photosynthesis processes, 10% is reflected, 2% is emitted as fluorescence, and the rest is transmitted (Lambers and Oliveira, 2019). However, this balance can vary depending on the chlorophyll content and concentration throughout the plant developmental phases, which itself is subject to environmental factors that influence physiological responses like growth, structural changes, and stress. The importance of Chl quantification, beyond its inherent ecosystem value, is widely documented in the agricultural literature, with efforts exploring its role in underpinning gross primary productivity (Houborg et al., 2015a), leaf nitrogen monitoring (Schlemmer et al., 2013), assessing health status (López-López et al., 2016), supporting fertilization management practices (Gabriel et al., 2017), and senescence detection (Noodén et al., 1997). Despite the importance of chlorophyll for phenotyping and agricultural purposes, accurately quantifying its temporal dynamics at different spatial scales (i.e., leaf, canopy, or field) remains a significant challenge, given the laborious and time-consuming sampling procedures required for its accurate characterization. From the diversity of methods available for examining leaf chlorophyll content, two of the most widely used include a destructive laboratory procedure based on *in vitro* spectrophotometric techniques (Wellburn, 1994; Porra, 2002; Netto et al., 2005) and a non-destructive method based on *in-situ* observations collected *via* chlorophyll meters, such as the Soil Plant Analysis Development (SPAD) system (Yuan et al., 2016; Shah et al., 2017; Dong et al., 2019).

However, despite the high accuracy provided by the laboratory method, or the portability offered by handheld sensors, both procedures face limitations when covering large study areas, where numerous samples are required to assess entire plant populations. An alternative and complementary approach to tackle this limitation is through combining field-based sampling (or scouting) with remote sensing based observations. Total chlorophyll can be tracked by its reflectance response using optical sensors (Curran, 1989), which can detect spectral absorption peaks within the visible wavelengths, centered at the 400–450 nm range and around 680 nm for Chl-a, and at the 450–500 nm range and around 650 nm for Chl-b. In recent decades, progress has been made in using multispectral satellite observations in combination with field data to estimate a range of “greenness” indices and gross primary productivity using MODIS (Wang et al., 2020), Landsat (Croft et al., 2015; Houborg et al., 2015b) and Sentinel-2 (Clevers and Gitelson, 2013; Delloye et al., 2018) platforms. Other initiatives have explored space-borne hyperspectral imagery from the EO-1 Hyperion sensor, tracking yield dynamics based on Chl content and leaf area index (Wu et al., 2008; Houborg et al., 2016). However, recent advances in miniaturizing optical sensors and systems, which can capture high spatial, spectral and temporal resolution data, offer new research opportunities to progress open questions in retrieval models and dynamics of Chl at both leaf and canopy scales. For example, studies based on unmanned aerial vehicles (UAVs) coupled with hyperspectral cameras have examined pigments content estimation (Zarco-Tejada et al., 2013a) by replicating modeling approaches already implemented with satellite and airborne-base data. With the enhanced spatial and temporal resolution afforded by such systems (Aasen et al., 2018), these technologies also bring new challenges in terms of the computational efficiency required to process, model, and analyze the large volumes of data collected.

Translating these massive quantities of hyperspectral imagery and *in-situ* data into useable information and knowledge requires improved and targeted modeling strategies. Early studies using UAV-based imaging spectroscopy were often focused on monitoring and characterizing croplands, retrieving Chl content, and other specific physiological properties using a range of methods. Broadly speaking, these approaches can be grouped into parametric, machine learning (ML), radiative transfer models (RTM), or hybrid methods (see Verrelst et al., 2019 for a full review). Parameterized relationships between spectral bands sensitive to physiological traits, more generally referred to as vegetation indices (VIs), are probably the most common approach to map pigments content (Haboudane et al., 2002), with examples including the Photochemical Reflectance Index (PRI; Zarco-Tejada et al., 2013b), the optimized soil-adjusted vegetation index (OSAVI) and the modified chlorophyll absorption in reflectance index (MCARI; Domingues Franceschini et al., 2017), among many others. Statistical regression approaches are routinely employed to capture relationships between spectral features and biophysical traits. For example, one of the more widely used linear methods is partial least squares regression (PLSR), which has been implemented to simultaneously estimate Chl and LAI (Kanning et al., 2018). ML regression algorithms have become increasingly popular due to their diversity of model types and utility for analyzing large datasets, with examples including random forest, and support vector machines. Bayesian algorithms, such as the Gaussian process regression (GPR; Rasmussen and Williams, 2006; Verrelst et al., 2012; Camps-Valls et al., 2016), have gained popularity in remote sensing applications due to their capacity to measure uncertainty and include prior knowledge about the modeled variables by using kernel functions. Together with GPR, an ensemble of multiple algorithms (Feilhauer et al., 2015; Vanbrabant et al., 2019) has been shown to outperform what can be achieved from application of any single method. Finally, more recent developments have sought to combine elements from the approaches mentioned above, resulting in hybrid methods that have the advantage of complementing the biophysical properties of VIs and RTMs with the computational efficiency and flexibility of non-parametric models, especially when dealing with large datasets (Capolupo et al., 2015). Hybrid-combinations remain an open and promising research path for phenotyping at canopy and leaf–level, with applications including training ML regression approaches with simulated VIs retrieved by RTMs (Liang et al., 2016; Houborg and McCabe, 2018) or producing ensembles of dimensionality reduction (DR) and MLR methods able to filter critical spectral predictors (Rivera-Caicedo et al., 2017; Shah et al., 2019) to boost hyperspectral derived results.

Machine learning has shown considerable potential for delivering novel insights in leaf Chl retrieval, yet there are numerous implementation challenges that can frustrate application, including algorithm choice, training data, learning strategies, and predictors selection (Verrelst et al., 2019). Identifying the right algorithm among the many available depends on evaluating elements such as accuracy, interpretability, complexity, scalability, and computational cost: pre-analysis steps that are not always followed. Some approaches make particular assumptions about the data structure (i.e., distribution), demanding an exhaustive exploratory data analysis prior to modeling. Establishing a learning strategy has important implications for making the most of limited training data and prediction purposes. For instance, a data integration strategy for multi-temporal observations is required to understand how dynamics in Chl content combine to affect spectral responses. Likewise, the idea of strengthening the predictive power by using hundreds of spectral bands as predictors may result in computationally expensive models and multicollinearity issues, thus requiring coupled DR methods or testing transformed variables that bolster the spectral features sensitive to Chl (i.e., VIs). Overall, although there is not a generic recipe that can be applied to most ML problems, a modeling framework that integrates the above-highlighted aspects provides a much-needed road-map for retrieving biophysical variables from hyperspectral data.

The present study aims to assess the robustness of a machine learning framework to map a metric of leaf-Chl through the use of multi-temporal ultra-high spatial resolution (e.g., order of millimeters) UAV-based hyperspectral imagery. This is done using a training dataset composed of multi-temporal *in-situ* SPAD observations, together with VI estimates from field-based spectra measurements (350–2,500 nm) at the leaf level. Coincident and high-spatial resolution UAV-based hyperspectral scans (400–1,000 nm across 272 continuous bands) were also collected to provide a spatially distributed extension to the point scale *in-situ* training collections. A novel aspect of this work is that the modeling framework provides strategies for selecting the best-suited training/retrieval combinations based on accuracy assessment, using multiple learning models, spectral bands, and VIs predictors, and performed under both sequential and retraining learning techniques. Three particular research objectives are explored in this study: (1) examining the ability of different training strategies (retraining versus sequential) to capture and exploit temporal correlations in leaf-Chl; (2) quantify any potential gain (*via* a feature selection method) from using pigment-based VIs as Chl content predictors versus individual spectral bands; and (3) evaluate the performance of different ML regression approaches to accurately model and retrieve a SPAD-based Chl metric under dynamic training and field conditions.

## Materials and Methods

### Study Area and Experimental Design

**Figure 1:**
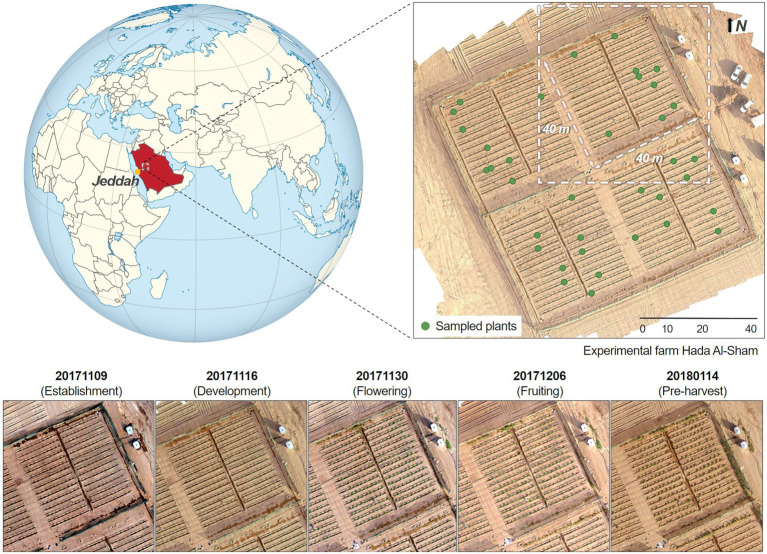
Temporal maps of the studied quadrant at the Hada Al-Sham experimental facility (Lat. = 21.797°, Long. = 39.725°), where a wild tomato species was cultivated. Five different collections were performed between November 2017 and January 2018. The globe map is part of a series of SVG locator maps of countries including elements that have been adapted from the file: Afro-Eurasia on the globe (red).svg, and distributed under CC-BY-SA-3.0 license.

As part of a phenotyping study of a wild tomato (*Solanum pimpinellifolium*) crop, data were acquired over an experimental farm located in the valley of Hada Al-Sham, approximately 250 m above sea-level and 60 km east of Jeddah, Saudi Arabia (Figure 1). The regional climate is tropical and subtropical desert, with annual rainfall averages of around 100 mm. Although there was no rainfall during the study period, several sandstorms occurred through the growing cycle. 1,200 individual tomato plants were planted across the field, spaced equally at 1.5 m intervals and comprising 60 rows aligned along the north-east direction at approximately 2 m separation. The area was divided into four plots, containing a total of 300 plants each. The substrate was predominantly sandy loam soil. Five field campaigns were conducted between the fall and winter seasons (from November 2017 to January 2018), capturing crop growth stages corresponding to establishment, development, flowering, fruiting, and pre-harvest. As the primary purpose of the original phenotyping study was to identify salinity tolerance within the chosen selections, the experiment included duplicate saline and freshwater irrigation across four sub-areas (see Barreto et al., 2019 for further details). Here we focus our analyses on a single quadrant in order to reduce the computational burden involved in multi-quadrant processing (considering the terabytes of imagery involved). Ultimately, results can be expanded to the rest of the field to explore the impacts of salt-stress.

### Field Spectra Data Collection

During each campaign, field-based reflectance spectra were collected close to solar noon using an ASD FieldSpec-4 (Analytical Spectral Devices Inc., Boulder, CO, United States) spectroradiometer, which samples data in the visible (VIS) and shortwave infrared (SWIR) spectral range (from 350 nm to 2,500 nm), with a resampled spectral resolution of 1 nm. From the total population of 1,200 plants, 36 individuals were randomly selected, and the reflectance response from three of their top leaflets measured (i.e., 108 samples for each campaign). Eleven sampling plants died before the last campaign (20180114, pre-harvest) due to strong winds, reducing the number of samples from 108 to 75 leaflets. An 8-degree fore optic lens was attached to a pistol grip to limit the field of view (FOV) diameter to 1.5 cm, measuring at a constant 10 cm zenith distance from each leaflet, which was placed on a black background. A white spectralon reference panel was used to calibrate the spectral measurements during the collection process. Five reflectance measurements were recorded for every leaflet, averaged, and spectrally resampled from 400 nm to 1,000 nm to match the spectral resolution of the UAV-based hyperspectral imagery (272 bands; see “Hyperspectral Imagery Collection and Calibration”) by using a Gaussian model based on the FWHM spacings and wavelengths information from the hyperspectral camera in the software ENVI.

### Ground-Truth Data Sampling

Non-destructive measurements of relative chlorophyll content (Chl) per leaf surface area were collected from the same spectrally sampled leaflets each day between 9:00 and 11:00 am local time, using a handheld SPAD-502 optical chlorophyll meter (Konica Minolta, Inc., Osaka, Japan). The operation of the SPAD meter is based on light transmittance at red and near-infrared wavelengths through a plant leaf. The instrument has two LEDs, one of which emits red radiance at 650 nm, with the other emitting near-infrared radiance at 940 nm. Most of the red light is absorbed by plants for photosynthesis, whereas longer near-infrared light passes through the leaf or is reflected. The ratio of transmittance at the near-infrared and red wavelengths is estimated and expressed as a unitless indicator, commonly referred to as SPAD units, which can range between 0 and 50 under standard measurement conditions (relative humidity <85% at <35°C) with a ± 1 unit accuracy, and up to 70 under high humidity/temperature conditions, with a drift of ±0.04 units per °C (Konica Minolta, 2009). Several studies have demonstrated near-linear and mostly exponential correlations between SPAD values and leaf chlorophyll content, although they can vary among species and growth habit groups (Markwell et al., 1995; Uddling et al., 2007; Cerovic et al., 2012; Parry et al., 2014; Shah et al., 2017). Since chlorophyll is not uniformly distributed in leaves and the device covers a small area of 6 mm^2^ (2 ×3 mm) per measurement, the SPAD average of five different locations across each leaflet surface was considered as a metric of its chlorophyll content.

### Hyperspectral Imagery Collection and Calibration

Spatially dense hyperspectral imagery was collected using a Nano-Hyperspec (Headwall Photonics, 2020a) push-broom camera integrated onboard a DJI Matrice 600 (M600) hexacopter (DJI, 2020). The Nano-Hyperspec was fitted with a 12 mm lens that afforded a horizontal field of view (FOV) of 21.1°, and collected data across the 400–1,000 nm spectral range in 272 continuous bands, with a 6 nm full-width half-maximum (FWHM). Flights were performed close to solar noon under clear sky conditions for all campaigns (see Figure 1 for specific dates), with a view zenith angle of zero and at an altitude of 15 m above the ground. Raw data were translated into radiance values using Headwall’s SpectralView package (Headwall Photonics, 2020b), including the specific sensor calibration files for each band (Barreto et al., 2019). Automated georectification and mosaicking were performed to obtain geometrically accurate data-cubes with a ground sampling distance (GSD) of 0.007 m. Further details on the geometric calibration process can be found in (Angel et al., 2020). For the spectral calibration, at-sensor radiance data-cubes were converted into surface reflectance by performing an empirical line correction method (Wang and Myint, 2015), which estimates a linear regression for each band, matching ground-truth reflectance with its correspondent radiance spectra in the hyperspectral image. Following the procedure of (Barreto et al., 2019), reflectance data were collected from six near-Lambertian gray-scale panels (60 ×80 cm) placed in the middle of the field before each hyperspectral scanning. The ASD FieldSpec-4 bare fiber optic (25°) was attached to the pistol grip measuring at a constant 50 cm zenith distance, limiting the FOV diameter to approximately 22 cm. A chess-patterned target and soil reflectance measurements taken from across the field were also used for validation. Finally, reflectance mosaics were spectrally enhanced by applying a pixel-based Savitzky–Golay smoothing filter (Savitzky and Golay, 1964) and running a de-noising process with the minimum noise fraction (MNF) transformation approach (Green et al., 1988), in order to attenuate any artifacts that may lead to distorted spectra shapes affecting the data reliability (Ruffin and King, 1999).

### Extraction of Vegetation Indices

**Figure 2:**
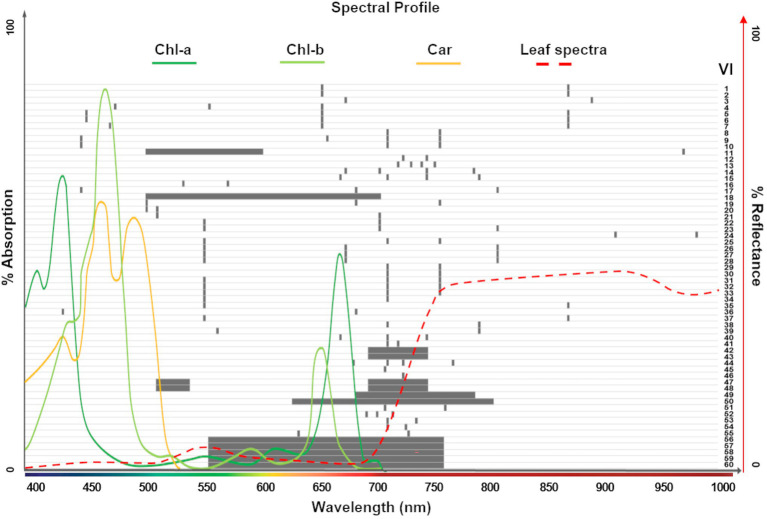
Spectral ranges covered by the 60 vegetation indices (VIs) explored in this study (gray bars; see Supplementary Table 1 for complete list). The absorption spectrum for photosynthetic pigments (Chl-a, Chl-b, and Car; dark green, light green and yellow lines, respectively; Lambers and Oliveira, 2019) and the typical leaf reflectance spectra (red dashed line) overlapping these ranges show the dominant pigments considered for each index. A total of 145 unique spectral bands were used in the formulations of the 60 VIs.

Vegetation indices (VIs) are mathematical formulations of spectral bands that are widely used to quantify structural, physiological, and biochemical plant characteristics. These relationships are based on established correlations between reflectance spectra features and specific phenotypic traits. In addition to band specific ratios, narrowband (or hyperspectral) VIs often combine many continuous bands to capture spectral profile features, such as slopes, curvatures, and absorption depths (Thenkabail et al., 2019). Our study investigates 60 significant VIs that have previously been reported in the hyperspectral literature to be correlated with leaf chlorophyll content at various stages. For instance, from the Index DataBase (Henrich et al., 2009; one of the most comprehensive online resources) we selected VIs that were reported in studies conducted with spectrally similar sensors (i.e., covering the 400–1,000 nm range, including CASI550 and PHI, with 288 and 244 bands, respectively). We also include the Chl indices used by (Zarco-Tejada et al., 2019) to generate large-scale chlorophyll content maps, as well as the summary of derivative VIs in (Thenkabail et al., 2019). In addition, the VIs explored by (Shah et al., 2019) to retrieve leaf Chl in wheat, and those studied by (Houborg et al., 2016) to detect leaf Chl dynamics from hyperspectral satellite imagery were added. In total, 145 unique spectral bands were used in the formulations of the 60 indices, which were arranged into nine groups based on their calculation of similar phenotypic properties (see Supplementary Table 1, including formulations and key citations, and Figure 2). The field spectral profiles and the hyperspectral imagery were used to calculate the VIs at a leaf scale and pixel level, respectively.

## Machine Learning Modeling Workflow

In this study, a variety of regression approaches were evaluated under a proposed machine learning framework for multi-temporal mapping of leaf chlorophyll content (in SPAD units). The retrieval process combines five steps, including feature selection, learning different methods, cross-validating each model, assessing their performance, and mapping the SPAD predictions. SPAD data described in “Ground-Truth Data Sampling” are used as the response variable *y*, and the predictor variables *x* are derived from the field spectra samples (“Field Spectra Data Collection”). Two training strategies are tested: one considers sequential learning, and the other a time-series or retraining prediction (Dietterich, 2002). In sequential learning, the entire sequence of ground-truth observations is used to train each model and make all the predictions. In retraining prediction, models are cumulatively trained or retrained, and predictions are retrieved with the sampled data starting from the first stage *t*_1_ up to a time *t*_1_ (i.e., *i* = 5 growth stages). Retraining implies a repetition of the workflow that generated the previously fitted model, but based on a new training dataset that reflects the most recent and current status of the plants, which is composed of the previous data and the new data (i.e., *t*_1_, *t*_1_ + *t*_2_, *t*_2_ + *t*_3_, *t*_3_ + *t*_4_, *t*_4_ + *t*_5_), thus re-fitting the model while keeping its underlying architectural components (i.e., predictor variables, hyperparameters). This is an important strategy to explore because models can be retrained progressively with newly sampled data.

**Figure 3:**
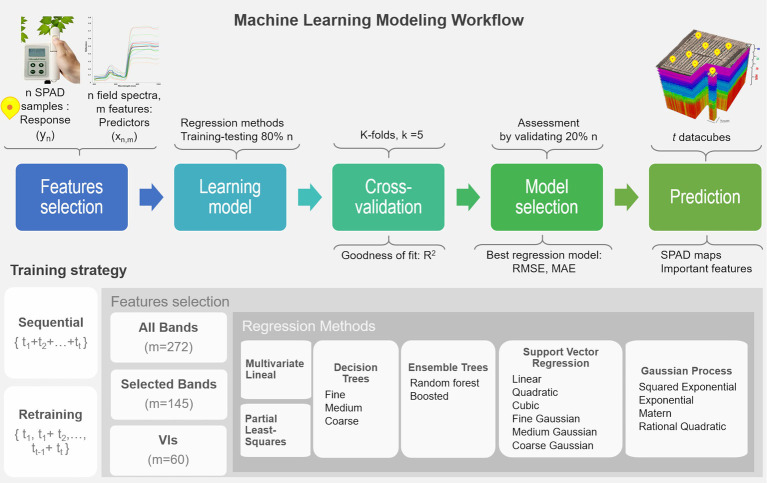
Machine learning workflow for the retrieval of multi-temporal leaf chlorophyll dynamics using ultra-high-resolution UAV-hyperspectral imagery. The retraining loop should operate using the selected algorithms by starting from the first dataset (i.e., *t* = 1), then re-running the fitting process on the next training datasets, but using the previously fitted predictor variables and hyper-parameters (i.e., *t*_1_ + *t*_2_, *t*_2_ + *t*_3_). In this way, the model is updated as new training data is used in the learning process, and predictions are estimated accordingly.

The learning workflow (see Figure 3) starts with the selection of predictor features, where each model is trained by using either all spectral bands (272 bands) or the set of vegetation indices (60 VIs), derived from the field spectra samples, allowing an investigation of the correlation and relevance of these variables as predictors. In addition, the subset of 145 bands that are used in the calculation of the various VIs were considered to examine any gain from transforming the spectral bands into VIs and the capability of the models to capture relevant and unknown relations between these selected bands and SPAD levels. The framework is evaluated with the most common nonparametric ML regression methods reported in retrieving biophysical variables from remote sensing applications (Verrelst et al., 2019). It is worth noting that the word nonparametric does not imply the lack of parameters, but that such parameters are adjustable and can be tuned by minimizing the estimation error while training takes place. In order to identify an optimal model structure, a total of 17 algorithms from three main categories are trained (e.g., linear-based, decision tree-based, and kernel-based). These include multivariate linear regression, partial-least-square regression (PLSR), decision trees, ensemble trees, support vector machines (SVM), and Gaussian processes regression (GPR). Kernel-based methods allow for an exploration of different types of mathematical functions (or kernels) to model the unknown or non-explicit relationships in the input data under a specific kind of function. For instance, those most commonly used in Earth Observation (EO) studies include linear, polynomial, and radial basis function (RBF) for SVM, and covariance-kernels for GPR (e.g., exponential, rational quadratic, RBF, and Matern; Camps-Valls et al., 2016; See more details in Supplementary Material). The architecture of some of these nonparametric approaches have embedded automated dimensionality reduction (DR) mechanisms, or band analysis tools (BAT; Rivera-Caicedo et al., 2017), to select relevant predictors, which is critical when dealing with hundreds of variables. For instance, the PLSR reduces the predictors to a smaller set of uncorrelated components, while the decision and ensembles of trees rely on pruning strategies, and GPR implicitly infers the feature’s relevance from a length-scale parameter enclosed in the covariance functions. 80% of the input dataset is used to train and test each model under a 5-folds cross-validation routine, with their goodness of fit estimated using the *R*^2^ metric (1) The remaining 20% of observations are used to identify the best performing model by assessing two prediction accuracy metrics: the root mean square error (RMSE), (2) the mean absolute error (MAE), and (3) Using these metrics, the most accurate model per method is employed to retrieve the multi-temporal SPAD predictions for each data-cube at a pixel-level. Since the pixel size is in the order of millimeters (e.g., 7 mm), the spectral profile of a pixel vector is assumed at a leaf scale. Using a plant delineation mask to exclude soil background (see Barreto et al., 2019 for further details), the best overall ML models are feed with the masked datacube to retrieve the SPAD maps and determine the most relevant predictors.


(1) $$R^{2}=1-\frac{\sum_{i=1}^{n}{\left(y_{i}-{\hat{y}}_{i}\right)}^{2}}{\sum_{i=1}^{n}{\left(y_{i}-\hat{y}\right)}^{2}}$$

(2) $$RMSE=\sqrt{\frac{1}{n}\overset{n}{\underset{i=1}{\sum }}{\left(y_{i}-{\hat{y}}_{i}\right)}^{2}}$$

(3) $$MAE=\frac{1}{n}\overset{n}{\underset{i=1}{\sum }}|y_{i}-{\hat{y}}_{i}|$$

In addition to retrieving SPAD maps, another useful output to examine from the selected methods is the importance of the features estimated by each approach while fitting the models. Feature selection approaches used for dimensionality reduction (DR), allow for the determination of each predictor’s relevance in any particular model by scoring the features with a relevancy metric. They also help to better understand the dynamics between dependent and independent variables and enable a subset of less redundant features that could lead to model improvement (Mladenić, 2006).

## Results

### Exploratory Input Data Analysis

**Figure 4:**
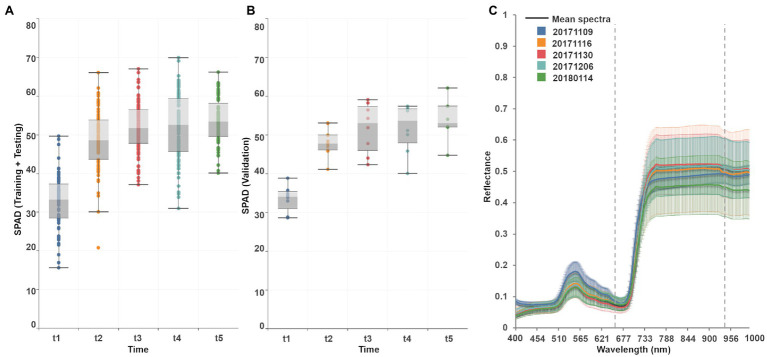
**(A)** Traditional boxplots show the multi-temporal distribution of SPAD observations divided into training/testing (left) and validation sets (right). Median values are shown as the horizontal lines near the box centers, and the quartiles are delimited by the horizontal lines above and below the median. Whiskers indicate the variability outside upper and lower quartiles, locating the maximum and minimum scores at the top and bottom ends, respectively. An outlier observation (21 SPAD units) is shown during the second campaign (*t*_2_) in the training/testing dataset. **(B)** Reflectance profiles collected per field campaign (format YYYYMMDD). The continuous lines indicate the mean spectra, and the filled areas define the range of reflectance measured each time. Dashed lines indicate the wavelengths where reflectance data reaches the lowest (~650 nm) and the highest (~950 nm) variability.

The various modeling trials performed in this study, as outlined in the workflow described in Figure 3, evaluate both sequential and retraining learning strategies. Ground-truth data comprise a total of 108 observations of SPAD samples and leaf spectra per field campaign, randomly split into two subsets: training/testing (80%) and validation (20%), assuring their distributions are as similar as possible. SPAD observations, considered as the prediction response, are variable, symmetrically distributed, and rising across the growing season (Figure 4A). For the first date, 50% of the data ranged between 29 and 38 SPAD units, with a median of 33. Half of the samples reached a higher median of 49 for the second date, within a range of 45–54 SPAD units. For the third campaign, the observations have a narrower distribution than the previous collection, with a minimum and maximum value of 38 and 67 units, respectively, although reaching a slightly higher median of 52 units. SPAD data for the fourth campaign were more widely distributed, with half ranging between 48 and 59 units, with a median value of 53 units. For the last date, observations were less variable, spanning between 40 and 66 SPAD units, with a median of 55 units. Although training/testing and validation datasets show a slightly different distribution, their median values follow a similar trend over time (Figure 4A).

Each of the 272 bands from the resampled field spectra data are considered as an individual prediction feature. Figure 4B shows the multi-temporal spectra mean and their standard deviation, which in general follow a similar pattern in the blue (450–510 nm) and red edge (660–730 nm) regions, although differing along the green (510–660 nm) and near-infrared (740–1,000 nm) wavelengths. For the first date (20171109), the maximum average green and NIR reflectance reached 18 and 49%, with a standard deviation of 2 and 3%, respectively. For the second, third, and fourth campaigns, the mean green peak decreased to ~15%, with a standard deviation of ~3%, and the mean NIR response increased to ~50%, with a standard deviation of ~10%: although the NIR response during the second date is up to ~13% higher. For the last date, green reflectances were similar to previous collection times, yet the average NIR responses dropped to 45%, with a standard deviation of ~10%.

**Figure 5:**
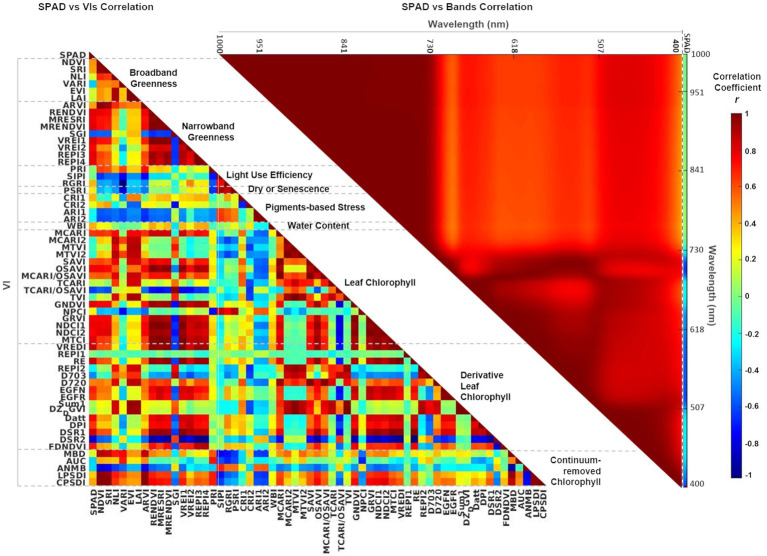
Correlation matrices showing Pearson’s *r* coefficient between SPAD and individual VIs (left-hand side), and between SPAD and individual spectral bands (right-hand side). The first column in the SPAD versus VIs matrix (left) and the last column in the SPAD versus Bands matrix (right) show the coefficients for the dependent variable SPAD. The color scale represents correlation coefficients between 1 and − 1. VIs are grouped and organized according to Supplementary Table 1.

Pearson correlation matrices were calculated to visualize any multicollinearity in both predictor sets, bands and VIs (Figure 5). In the case of spectral bands, SPAD data has a low negative correlation (−0.2 < *r* < −0.3) with bands in the blue (400–510 nm) and red ranges (650–680 nm), but more highly negatively correlated (*r* < −0.6) with the green spectral window (510–650 nm) and the red-edge bands (680−730 nm). In contrast, near-infrared bands (730–1,000 nm) are weakly correlated (*r* ≈ 0) with SPAD, but with high multicollinearity between them (*r* > 0.8): recognized as the Hughes phenomenon, which is thoroughly documented in the literature (Thenkabail and Lyon, 2012). Concerning the VIs (Supplementary Table 1), the narrowband greenness indices have a stronger correlation with SPAD than the broadband greenness indices. In contrast, photosynthesis efficiency, senescence, and pigments–based indices are weakly correlated with both SPAD and the other indices. For the set of VIs that evaluate leaf chlorophyll based on reflectance and derivative spectra, most show a high correlation with SPAD, while several (10 out of 31) have low inter-correlation with the rest. Finally, the group of continuum-removed VIs exhibit a low correlation with SPAD, with two negatively correlated with the other indices. Overall, although some of the indices are strongly inter-correlated (*r* > 0.8), this is only a measure of the association between them, not their causation. All the bands and VIs were included as predictor variables to let the MLR methods evaluate their relevance in predicting leaf chlorophyll.

### Multiple Model Regression Assessment

**Figure 6:**
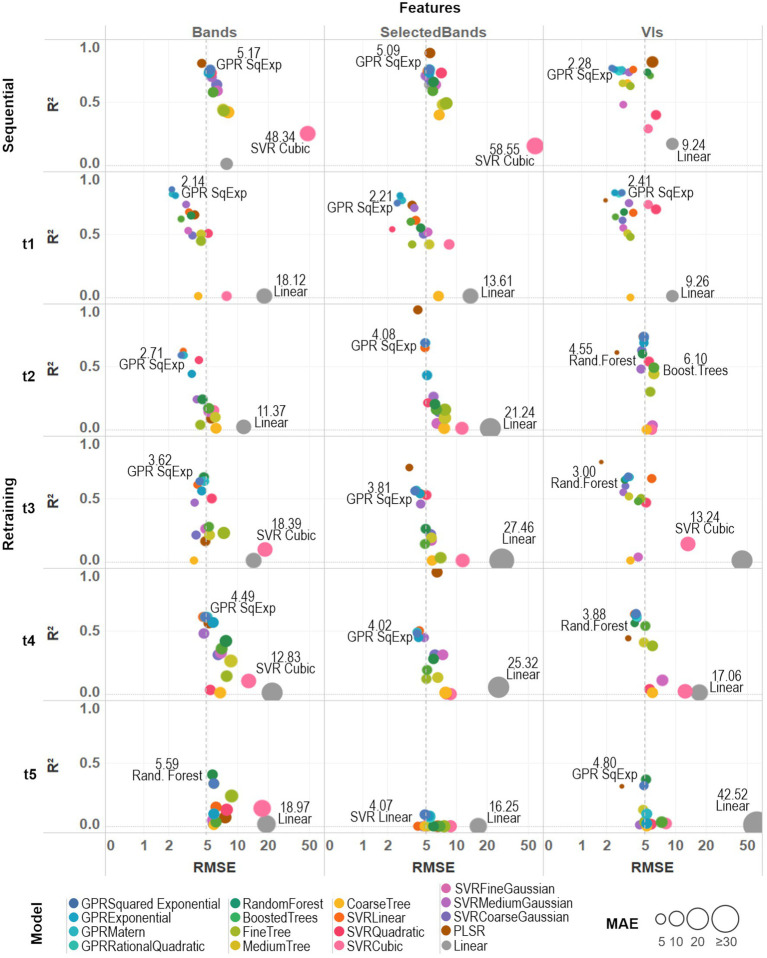
A comparative assessment was performed between the 17 trained models by comparing three different metrics: *R*^2^, RMSE, and MAE. Model-fit and accuracy were evaluated under both training strategies (sequential and retraining) and considering different sets of prediction features (all spectral bands, selected bands from VIs, and VIs). An average prediction error threshold of 5 SPAD units was established to evaluate individual model accuracy (dashed line). Base-10 log scale is used for the *x*-axis (RMSE).

Three different metrics, *R*^2^, RMSE, and MAE, were used to undertake a comparative accuracy assessment of the models. *R*^2^ was used to assess the performance of the models from the *k*-fold cross-validation, and RMSE and MAE to evaluate their actual accuracy by using ground-truth validation data. Figure 6 summarizes these metrics for all the algorithms tested under the two training scenarios and three sets of predictors: all bands, selected bands used for calculating the VIs, and VIs.

For the sequential strategy case, PLSR coupled with band predictors achieved the best fitted models, either using all the bands (*R*^2^_AllBands_ = 0.80) or the selected subset (*R*^2^_SelectedBands_ = 0.89); although retrieving more accurate results when using all of them (RMSE_SelectedBands_ = 5.45, MAE_SelectedBands_ = 4.27 > RMSE_AllBands_ = 4.40, MAE_AllBands_ = 3.50). As can be seen in Supplementary Table 2, the various GPR models, along with SVRLinear and SVRMediumGaussian, were the best-performing algorithms across both types of predictors, reaching low RMSE and MAE around 5 SPAD units when using spectral bands and below 3 SPAD units for the VIs case (and with an *R*^2^ above 0.7). In comparison, multivariate linear regression and SVRCubic models produced the poorest results, with higher errors (RMSE > 5, MAE > 6) and *R*^2^ below 0.3. Overall, the GPRSquaredExponential (i.e., with a squared exponential kernel) achieved the second-best scores when considering both types of prediction features (RMSE_AllBands_ = 5.17, RMSE_SelectedBands_ = 5.09, RMSE_VIs_ = 2.28, MAE_AllBands_ = 4.06, MAE_SelectedBands_ = 3.96, MAE_VIs_ = 1.98, *R*^2^_AllBands_ = 0.76, *R*^2^_SelectedBands_ = 0.76, *R*^2^_VIs_ = 0.77). In general, most of the models coupled with band predictors reached comparable *R*^2^ and accuracy results, except for PLSR and the multivariate linear approaches; whereas modeling with the set of VI predictors was more accurate and better fitted than performing with the spectral bands.

For the retraining strategy, accuracy and goodness-of-fit of the models were assessed date by date. Overall, improved RMSE and MAE metrics were achieved using band predictors when compared to the sequential strategy case, although the *R*^2^ decreased gradually over time (Supplementary Table 2). Results using VI predictors provided models that were comparable (i.e., similar *R*^2^) to those produced by band features, but also recorded a similar drop in *R*^2^ through time and producing slightly less accurate predictions. Particularly for the last campaign (*t*_5_), a considerable drop (*R*^2^ > 0.4 for all models) can be explained by the reduced number of spectral leaflet samples used to train/test the models (see “Field Spectra Data Collection”). For the first date (*t*_1_), the GPRSquaredExponential was the most accurate (RMSE_AllBands_ = 2.14, MAE_AllBands_ = 1.73) when paired with all the bands, and the best fitted (*R*^2^_AllBands_ = 0.86, *R*^2^_SelectedBands_ = 0.75, *R*^2^_VIs_ = 0.83) using the three sets of predictors: although beaten by PLSR when coupled with VIs (RMSE_VIs_ = 1.93, MAE_VIs_ = 0.86). For the second campaign (t2), the GPRSquaredExponential again produced the most accurate results from the all bands-based case (RMSE_AllBands_ = 2.71, MAE_AllBands_ = 2.13), while the PLSR model was better fitted using the selected bands (*R*^2^_SelectedBands_ = 0.95). In the VIs-based case, the PLSR was the most accurate (RMSE_VIs_ = 2.56, MAE_VIs_ = 0.98), but GPRSquaredExponential was better fitted (*R*^2^_VIs_ = 0.74). For the third date (t3), the GPRSquaredExponential model achieved the highest accuracy (RMSE_AllBands_ = 3.62, MAE_AllBands_ = 3.01) under the all bands-based setup, and RandomForest the best fitting (*R*^2^_AllBands_ = 0.67); whereas in the selected bands-based and the VIs-based case, PLSR was the best fitted (*R*^2^_SelectedlBands_ = 0.75, *R*^2^_VIs_ = 0.79), and the most accurate (RMSE_SelectedBands_ = 3.77, MAE_SelectedBands_ = 2.66, RMSE_VIs_ = 1.72, MAE_VIs_ = 0.78). For the fourth campaign (t4), the PLSR trained with the selected bands was the best-fitted model (*R*^2^_SelectedlBands_ = 0.97), although it was poorly accurate (RMSE_SelectedBands_ = 6.37, MAE_SelectedBands_ = 5.30) to the others performance. In contrast, the GPRSquaredExponential was the second best-fitted model (*R*^2^_AllBands_ = 0.61, *R*^2^_VIs_ = 0.64), reaching the highest accuracy when trained with all the band features (RMSE_AllBands_ = 4.49, MAE_AllBands_ = 3.99). However, this model was exceeded by the RandomForest (RMSE_VIs_ = 3.35, MAE_VIs_ = 1.53) when using VI predictors. For the last date (t5), the RandomForest model shown the highest accuracy (RMSE_AllBands_ = 5.59, MAE_AllBands_ = 4.37) and best-fitting (*R*^2^_AllBands_ = 0.41) when coupled with all the bands; whereas PLSR was the most accurate when trained with VIs (RMSE_VIs_ = 2.89, MAE_VIs_ = 0.96). Overall, sub-optimal results were retrieved using the selected set of bands, and all the models were poorly fitted with *R*^2^ below 0.1.

Under both training strategies, the three sets of predictors showed comparable performance, yet most of the VIs-based models achieved better scores than the band-based ones. Slightly better results were reached by most of the models when trained with all the bands than with the selected ones (i.e., between ~0.1 and 0.4 units in *R*^2^ scores), except for the PLSR. Also, despite being the weakest approach (with the highest RMSE and MAE, and the lowest *R*^2^) across the different strategies and predictors, the multivariate linear (or linear) model scores were considerably improved when using the subset of selected bands (see Supplementary Table 2; Figure 6). For instance, the linear model improved relative to the medium tree model performance and was comparable to the random forest model results under the sequential strategy. Considering the slightly better scores retrieved by the full set of spectral bands than the set of selected bands, the models trained with all 272 bands were considered together with the VIs-based ones to perform the predictions and select the best among them to produce the SPAD maps.

### Multiple Model SPAD Predictions

**Figure 7:**
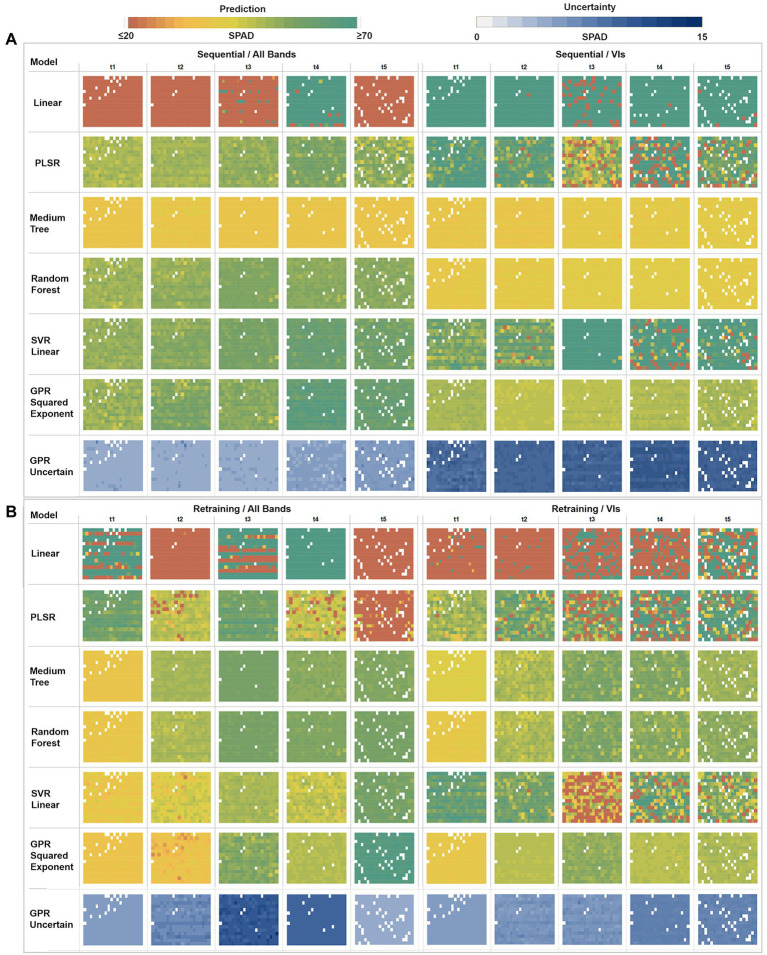
Multi-model comparison of averaged SPAD predictions and GPR uncertainties at a plant level. **(A)** Sequential strategy results using All Bands versus VIs. **(B)** Retraining strategy results using All Bands versus VIs. Each box comprises a matrix of cells representing the mean predicted SPAD per plant, following the field sowing arrangement (rows × columns).

Based on the assessment metrics, a criteria model selection was established to map the SPAD predictions. Different factors, such as leaf water content and irradiance changes, can introduce between 2 and 4 unit biases in SPAD readings (Martínez and Guiamet, 2004), in addition to the instrumental accuracy of ±1 units. Accounting for these influences and equating the quality of the SPAD predictions with the SPAD readings, we defined an error threshold of up to 5 units for both metrics (RMSE and MAE) under the assumption that all of the errors would have the same magnitude, which is the only theoretical case when RMSE and MAE would be equal. Under these criteria, models with average prediction errors above 5 SPAD units were excluded from the final selection, and the best-fitting model from each approach was used to retrieve the predictions from the hyperspectral imagery (see Figure 6). Accordingly, PLSR, the medium trees, random forest, SVR linear, and GPR squared exponential models were selected together with the linear regression, which was only included for comparison purposes despite it showing inferior performance among all model configurations. These trained models were used to retrieve the SPAD values at a pixel level on the multi-temporal hyperspectral data-cubes, then averaged at a plant level to evaluate the spatial and temporal distribution of the predictions across the study area. Figure 7 shows an array of the results organized by learning strategy, type of predictors, time, and types of models, where each box comprises a matrix of cells that represent the mean predicted SPAD per plant, following the same sowing arrangement of the field (rows × columns).

In the sequential strategy (Figure 7A), most of the models retrieved homogeneous SPAD maps across time, hence not showing significantly different changes during the growing season. Of note, the multivariate linear model produced results at the extremes when using different predictors, underestimating (≤ 20 units) when using bands, and overestimating (≥ 70 units) when using VI predictors. Similarly, PLSR estimates diverge under different predictor scenarios, realizing homogeneous result series (~30–50 units) through the band predictors while overestimating with VIs (≥ 60 units). In contrast, the medium tree model retrieved almost identical results for both cases, with SPAD values ranging around ~40 units: comparable with the performance of random trees when only using VI predictors. However, when using band features, random forest yielded similar results to SVR linear and GPR squared exponential models, with more variable retrievals between ~30 to 50 units (although the GPR-based map for the fourth date shows higher values around ~60 units). Using VI features, SVR-based results were more heterogeneous (around ~45 units) during the first two campaigns than the retrievals (≥ 60 units) during the last three dates. Overall, following a sequential learning strategy, GPR squared exponential predictions were the most dynamic across time, indicating its flexibility to learn and model temporal dynamics. However, this performance was degraded when using a more straightforward set of predictors like vegetation indices.

In general, for the retraining strategy (Figure 7B), the performance of the learning algorithms over both types of predictors was similar, with SPAD estimates varying over time and space - except those calculated by the multivariate linear regression and PLSR, which led to poor retrieval performance. Again, medium tree and random forest methods, combined with both predictor sets, reached matching results, increasing across time between ~30 and ~ 60 SPAD units during the first and last campaigns, respectively (although the VIs-based maps present slightly lower values than the bands-based ones). In the case of the SVR linear method, contrasting results were produced by each class of predictors, showing an ascendant trend across the time when using band features, comparable to the decision trees and GPR results. However, the SVR linear method underestimated results using VIs, even yielding values below 20 SPAD units for some plants during the last three campaigns (*t*_3_, *t*_4_, *t*_5_). In contrast, the GPR squared exponential results are congruent with the decision trees-based predictions under both types of predictors, although different estimates were achieved for the second and last dates. When using band features for the last campaign, the GPR-based map overestimated results, with values above ~60 SPAD units.

Together with the mean SPAD estimates, GPR reports the standard deviation at 95% (*σ_95%_*) confidence interval for each prediction, which is used as an uncertainty metric to assess the variance of the retrievals. For the sequential learning case (Figure 7A), homogeneous variances were achieved across the whole series, with low uncertainties between 4 and 6 SPAD units, when training with the band predictors, but high uncertainties ranging between 10 and 15 SPAD units when using VI features. In contrast, under the retraining strategy (Figure 7B), heterogeneous variance levels were observed throughout the series. For instance, an increase in the uncertainty was reported when using band features, starting from 5 to 6 units in the first date (20171109), then between 6 and 7 units during the second date (*t*_2_), until reaching standard deviations between 9 and 11 units during the third collection (*t*_3_). After this, the uncertainty levels decreased slightly to around 10 units on the fourth date (*t*_4_), achieving the lowest variance in the last date (*t*_5_) with 4 SPAD units. More stable variances were achieved using VI predictors, starting with low uncertainty levels between 5 and 6 SPAD units in the first three dates, with a minor rise of variance for the last two dates, with values between 6 and 8 units.

### Model Selection

**Figure 8:**
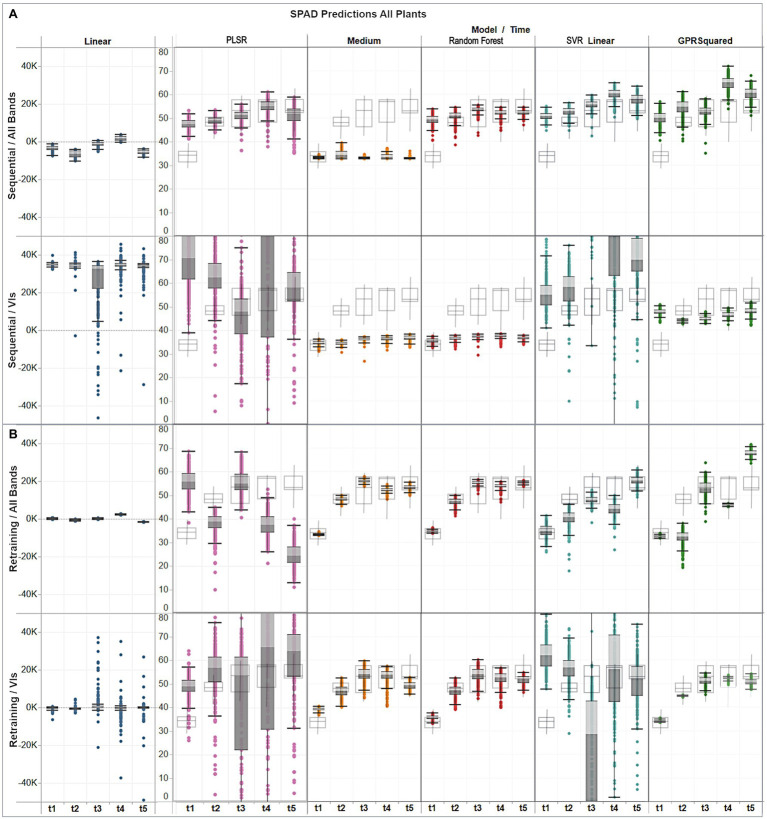
Assessment of multi-temporal predictions distribution (gray colored boxes) against the actual distribution (gray delineated boxes) of the validation data, showing **(A)** distribution per model under the sequential strategy using band and VI predictors; and **(B)** distribution per model under the retraining strategy using band and VI predictors.

The identification of suitable learning algorithms and predictors is a critical step to develop accurate SPAD retrieval maps. Therefore, an additional evaluation of the previously selected methods was performed by comparing the retrieved and original distributions of the validation dataset. Figure 8 presents the variability of SPAD predictions and *in-situ* collected measurements used for validation, with colored and gray delineated box-plots, respectively. As can be seen, the multivariate linear regression results were widely dispersed, either overestimated or underestimated, and divergent from the measured data distribution. In the case of PLSR, the distribution of the estimates reflected the validation reference only under the sequential training strategy using band predictors. Overall, most of the pre-selected algorithms achieved relatively consistent results, except SVR linear when combined with VI predictors.

For the sequential approach (Figure 8A) with band features, predictions from all models, except for the medium trees, followed the ascending trend across time observed in the *in-situ* data, although exceeding them by up to 15 units on the first date, if comparing their medians. The best distribution matches with the observations were obtained by random forest, and GPR squared exponential only on the third date (*t*_3_). In contrast, when using VI predictors, overall median estimates were below the ground-truth data distribution by between 10 and 20 SPAD units for the decision trees models, and between 3 and 10 units for the GPR model.

For the retraining case (Figure 8B), the performance was generally higher relative to the sequential strategy and more consistent between the assessed algorithms. In particular, the distribution of the results from the medium trees and random forest were comparable, reaching similar medians, although with different dispersion, over the different campaigns. The GPR squared exponential model achieved better results when combined with VI predictors, following the SPAD observations trend, although with less dispersed distributions and reaching median values between 1 and 5 SPAD units below the *in-situ* data. The best predictions were achieved by combining random forest with VI features, retrieving minor discrepancies between 1 and 3 units in the median values compared to the SPAD measurements, and yielding matching spread distributions, as shown by the minimum and maximum values of each campaign dataset.

Based on the accuracy assessment and the analysis of the prediction distributions achieved by all the evaluated methods, three out of the 17 regression models were selected to retrieve the multi-temporal SPAD maps at a pixel scale. The selected methods included the PLSR using all bands under the sequential strategy, and random forest and GPR squared exponential, using vegetation indices as predictor variables under the retraining strategy.

### Multi-Temporal Spatial Predictions of SPAD

**Figure 9:**
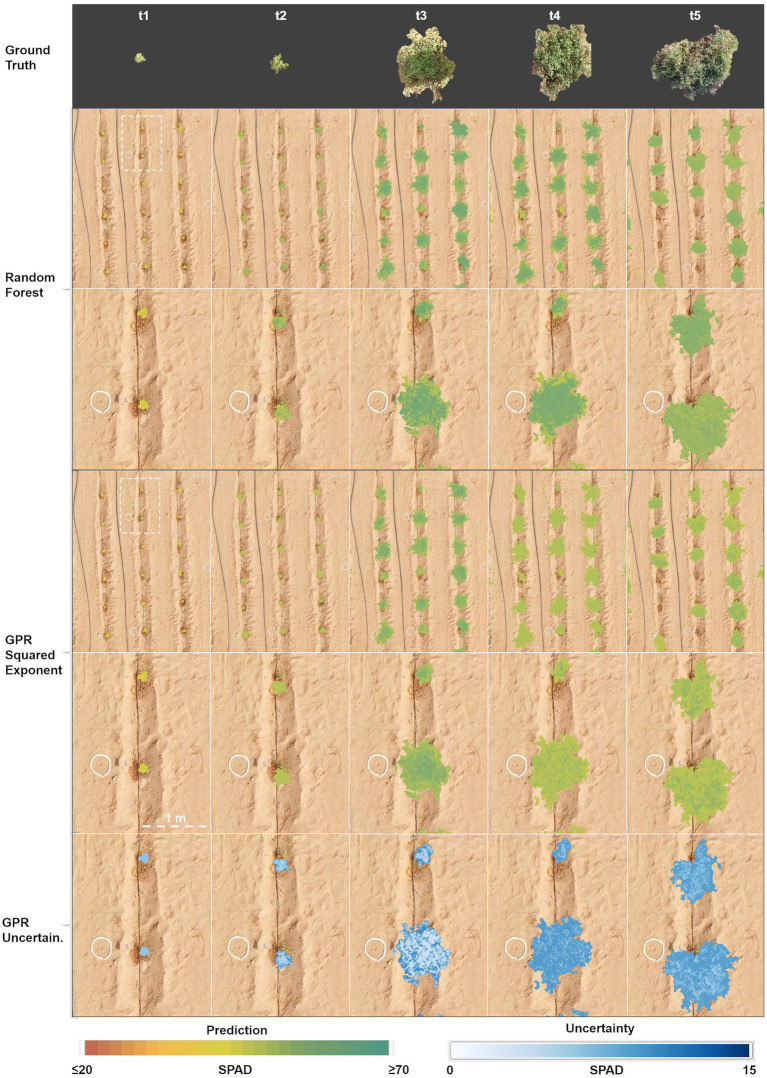
Comparison of multi-temporal SPAD prediction maps generated with PLSR coupled with band predictors, random forest and GPR squared exponential models, using vegetation indices predictors (lower panels). True color-balanced pictures of a showcase plant (upper panel) depict changes throughout the growing cycle. The gradient-colored bars represent the estimated SPAD values in the range between 20 to 70 units, with a bin size of 2. The SPAD uncertainties are shown in the range between 0 to 15 units, with a bin size of 1.

The hyperspectral mosaics, and the vegetation index data-cubes derived from them for each campaign, were used as input to feed the three selected regression models and to retrieve the multi-temporal SPAD maps at a pixel scale. Figure 9 depicts a comparison of the results achieved by each method over some of the sowed furrows, showcasing the differences between PLSR, random forest and GPR squared exponential estimates throughout the study period. For the first three campaigns, the PLSR model reached different results than the other two methods, with a slight increase from an average of 48 units in the first stage (*t*_1_) to 50 units in the third (*t*_3_), whereas random forest and GPR squared exponential reached similar estimates, with an increase in SPAD values from an average of 30 units in the first stage (*t*_1_) to 55 units in the third (*t*_3_). The PLSR and GPR-based estimates were uniformly distributed in leaves, changing over time without marked differences between stages, especially during the last two dates. However, some negative retrievals from the PLSR approach during the third and fourth campaigns can be seen as gap pixels in the showcased plants in Figure 9. On the other hand, random forest-based predictions differed from time to time, with a slight decrease in the last stage and showing more clustered estimates toward the center of the plants surface.

The plant growth dynamics can be described from the multi-temporal SPAD retrievals detailed in Figure 9. As can be observed, leaf chlorophyll content increases as plants grow and increase their leaf density from the establishment stage (*t* = 1), reaching a maximum value at the start of flowering (*t* = 3). Plants reach a mature state and produce fruits (*t* = 4), where SPAD estimates from PLSR increase slightly, whereas random forest results remain at the same level, and retrievals from GPR decrease slightly. At the pre-harvesting stage (*t* = 5), leaves and stems gradually age, turning yellowish, which is evident from the low SPAD levels predicted by all the methods. These dynamics are consistent with the distribution analysis described previously (Figure 8B), and illustrate how SPAD results from the three methods follow a similar temporal trend, albeit with GPR retrievals presenting a more homogeneous and tighter distribution than PLSR and random forest estimates. The uncertainty maps retrieved by the GPR algorithm were plotted to assess the variance of the predictions at the pixel level. As previously noted, the uncertainty of the estimates varies across the map series, starting with low (~4–6) standard deviations during the first three stages and slightly higher values during the fruiting and pre-harvesting phases (~6–8 units). The uncertainty of the predictions could come from either the propagation of uncertainties through time or high variations in the leaf-Chl levels’ dynamics. Moreover, when zooming into the maps at a plant level, the spatially distributed uncertainty levels can be observed over the plant projected areas, although with some higher variances associated with either bright or shadowed pixels.

### Important Features

**Figure 10:**
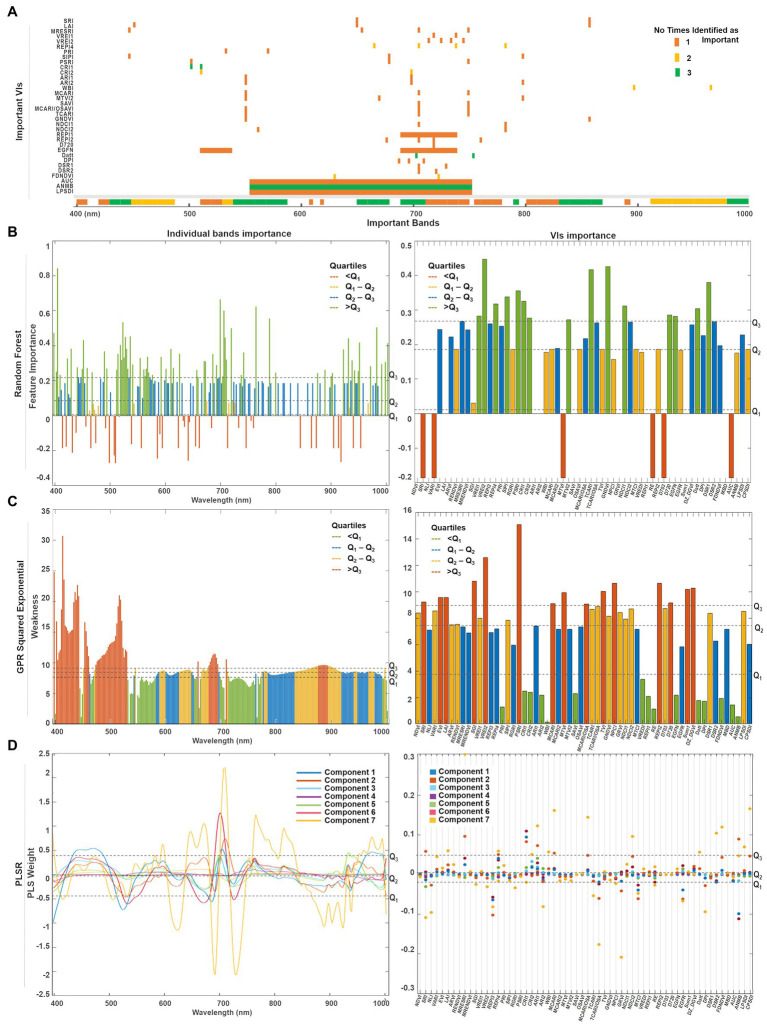
**(A)** Summary of the top predictors (bands and VIs) that were identified by either one (orange), two (yellow), or three (green) of the selected methods. A quartile classification was used to rank the top features of each method. **(B)** For random forest, the highest quartile was set as threshold to identify the most important bands (*Q*_3_ = 0.21) and VIs (*Q*_3_ = 0.27). **(C)** For GPR squared exponential, the lowest quartile was set as threshold to classify the less weak bands (*Q*_1_ = 8.02) and VIs (*Q*_2_ = 7.16). **(D)** The predictor weights were retrieved for the first seven components used to fit the PLSR model. The highest quartile was set as the threshold to denote the most relevant bands (*Q*_3_ = 0.48) and VIs (*Q*_3_ = 0.04).

The PLSR, random forest, and GPR approaches include feature selection mechanisms in their architecture to score the band and VI predictors base on their relevance toward the SPAD variable (see Supplementary Material). Various metrics are reported in each model: for instance, feature importance for the random forest, weakness index for GPR, and weight index for PLSR (Figure 10). The PLSR weights are retrieved for each of the components used to fit the model, which describes how strongly each component depends on the original predictors. The number of components (i.e., seven) was tuned by minimizing the error of the predictions through cross-validation during the training/validation stage (Figure 10D). The predictor scores were extracted from the three selected models when trained under the strategy they perform the best (Figure 8). Thus, the fitted models under the sequential strategy were used for comparing the spectral bands’ metrics, whereas their results under the retraining strategy were gathered for the VIs case.

Since each model has its own metric, their predictors’ scores cannot be directly compared. Thus, we use a quartile classification to rank the top features of each method, where each quartile contains 25% of the total predictors. For PLSR (Figure 10D) and random forest (Figure 10C), the highest quartile (*Q*_3_) was set as the threshold to denote the most important features, whereas the lowest quartile (*Q*_1_) was set for the GPR squared exponential (Figure 10B) to indicate the less weak (or most relevant) variables. A summary of the top predictors retrieved by either one, two, or three of the selected methods were highlighted (see Figure 10A), allowing the identification of those variables that might play a physically meaningful role in predicting Chl levels. The spectral information contained by the highlighted VIs can be traced in Figure 10A. Only three VIs contain information from the blue spectral region (~450 nm), followed by a small group of VIs that gather information from the green wavelengths (500 nm −550 nm). However, most of the important indices comprise the information from the red edge region (650–750 nm), and few individual VIs collect the information from narrow spectral ranges along the near-infrared (~800, 870, and 970 nm).

A combination of both individual bands and band ranges were identified as the most relevant for each model (Supplementary Table 3). Groups of continuous bands comprising less than 10 nm spectral range were counted as a single variable around the central band. For instance, the bands around 405 nm were identified as relevant by random forest and GPR, but weighted low by PLSR, although with a broader range for the random forest (i.e., 400–410 nm) where Chl-a is highly absorbed. The spectral variables between 450 and 490 nm were ranked as relevant by all the methods, coinciding with the most substantial Chl-b absorption. Bands from the green spectral range (i.e., 530 and 590 nm), where Chl reflectance peaks, were also highlighted as relevant by random forest and GPR, but not highly weighted by the first and seventh PLSR components. Toward the red wavelengths, random forest identified two narrow ranges (i.e., 610 and 620 nm), that agreed with GPR and PLSR in the Chl-a and Chl-b absorption crest (i.e., 650–670 nm). From 690 nm to 750 nm, the red-edge region was also highlighted as relevant by all models, although GPR identified two specific ranges: one spanning from 695 to 710 nm at the beginning of the red-edge, and another from 720 to 760 nm, where the red-edge inflection point shifts accordingly to Chl content. The three methods identified a critical thin region between 760 and 770 nm at the end of the red-edge. Near-infrared (NIR) bands indicated variable relevancy levels among the methods: PLSR highly ranked the region between 800 and 830 nm, while the flat sill on the plant reflectance spectra (i.e., 910–1,000 nm) was identified by random forest and PLSR, whereas GPR highlighted the very end of the NIR (i.e., 980–1,000 nm).

A total of 15 VIs were scored as relevant by each method (Supplementary Table 3; Figure 10). From the greenness indices, PLSR identified two of the broadband VIs (i.e., SRI and LAI) as significant and corresponding with random forest in selecting the narrowband red-edge position index REPI4. However, random forest highlighted two other indices of this category as relevant (i.e., VREI1, VRE2). The light use efficiency indicators (PRI and SIPI) were considered by GPR and random forest as critical, while the senescence index (PSRI) was only highlighted by random forest. In contrast, the stress-on-pigments production indices were highly scored by the three methods (i.e., CRI1, CRI2, ARI1, and ARI2). The water content index (WBI) was classified as relevant by the GPR and the PLSR models. From the 15 leaf Chl indices, random forest selected four of them (i.e., MTVI2, TCARI, GNDVI, and NDCI1), GPR selected just one (SAVI), while the PLSR model selected three (i.e., MCARI, MCARI/OSAVI, and NDCI2). From the derivative-based leaf Chl indices, all the methods highlighted the Datt index as critical, although random forest identified three more (i.e., D720, EGFN, and DSR1) and GPR identified another two (i.e., DPI and FDNDVI)—coinciding with PLSR in selecting the FDNDVI index. Finally, GPR and PLSR both identified ANMB from the continuum removed-based indices, whereas only GPR highlighted the area under the curve index (AUC) and the leaf plant stress detection index (LPSDI).

The feature importance analysis was extended to the models trained with the subset of 145 bands (i.e., the source of the VI predictors) to investigate how the feature selection operates on a smaller dataset. The same training strategies and ML approaches were used to retrieve, score, and classify the 145 selected bands, following the rationale presented previously (Supplementary Figure 1). Supplementary Table 4 summarizes the relevant bands and spectral ranges identified from the 145 bands subset.

## Discussion

### Sequential Versus Retraining Learning

Modeling physiological traits such as leaf Chl content throughout a crop growing season requires treating plant traits as continuous processes across time, which can be accounted for through implementing sequential and retraining learning strategies. Sequential learning is a common practice in remote sensing, wherein the full observed series is used to fit a single model assuming that the relationships between the prediction features and the independent variable remain fixed through time (Dietterich, 2002). In contrast, the retraining strategy uses a loop to learn a model progressively as new data is collected. Deciding whether to follow one or the other relies on the modeling problem and the data itself, since both are data-driven strategies after all. This study followed a simple and useful diagnostic suggested in the machine learning literature by examining the target variable distribution (Sculley et al., 2014).

Leaf chlorophyll and reflectance response change significantly through time, which is evident in this study by analyzing the distributions of sampled SPAD and spectral data (Figure 4A). Hence, the correlation between SPAD and predictors, either by bands or VIs, are dynamic as well. The temporal distribution of the SPAD validation dataset can be used as a reference to assess the coherence of the SPAD retrievals (Figure 8). In doing this, the accuracy metrics (MAE, RMSE) can be used jointly to determine the best candidate models to retrain (i.e., PLSR, random forest, GPR squared exponential). That is, the retraining loop should operate using the selected algorithms by starting from the first dataset (i.e., *t* = 1), then re-running the fitting process on the next training datasets (i.e., *t* = 2, *t* = 3), but using the previously fitted predictor variables and hyper-parameters. In this way, the model is updated as new training data is used in the learning process, and predictions are estimated accordingly.

The predicted SPAD maps averaged at a plant level and retrieved under the retraining strategy (Figure 7B) are coherent with the SPAD distributions of the *in-situ* validation dataset (Figure 8B). Such a result demonstrates the capability of the selected models to learn from *in-situ* data using a retraining routine, and thereby enhances the capacity to turn UAV-based hyperspectral imagery into valid multi-temporal SPAD maps. It also proves the capability and flexibility of the retraining strategy to capture temporal dynamics in chlorophyll levels from series of hyperspectral imagery by fitting multi-temporal regression models and advancing, for instance, uni-temporal approaches that develop individual growth-stage models (Aasen and Bolten, 2018). Such a strategy also offers a solution to open questions raised in some related studies, where sequential learning was implemented to map Chl series from satellite (Houborg et al., 2016) and UAV (Vanbrabant et al., 2019) hyperspectral images, and advising further investigation in learning regression approaches capable of capturing subtle temporal dynamics linked to short-term variations in plant traits.

Most of the selected algorithms performed significantly better under the retraining learning strategy, although the goodness-of-fit (*R*^2^) estimated from the training/testing dataset can be affected as new training data is introduced. Further experiments can be conducted to incorporate a time component (i.e., hyper-parameter, kernel) in the regression algorithms definition, which can be fully dedicated to capturing temporal correlations, and non-stationary behavior associated to Chl content dynamics. One candidate to consider for advancing more specialized modeling structures is the Gaussian process, which can be composed of temporal and spectral covariance kernels, as already demonstrated by other applications in modeling solar irradiation predictions (Camps-Valls et al., 2016).

### Leaf Chl Retrieval Using Spectral Bands Versus Vegetation Indices

Feature transformation is a critical task in any machine learning framework, especially when involving datasets comprising of hundreds of variables. This aspect should be carefully reviewed by evaluating the types of variables that are part of the dataset and exploring possible transformations and reductions to optimize the model performance (Guyon and Elisseeff, 2003). In this study, three elements can be highlighted regarding evaluating VIs as transformed variables out of reflectance spectral bands. First, using VIs slightly improves the goodness-of-fit and prediction accuracy of different types of ML models. Second, the VI predictors approach provide an alternative way to use spectral variables without affecting the capturing of temporal dynamics. Third, hyperspectral VI predictors add specific biophysical background to the training knowledge, hence enriching model interpretability.

Few studies in the literature have explored retrieving SPAD-based Chl levels using VI predictors derived from hyperspectral datasets. For example, linear regressions (Qi et al., 2020), random forest (Shah et al., 2019), or Cubist (Houborg and McCabe, 2018) have been combined with different types of VIs, reaching more accurate results than using spectral bands, which has also been achieved in this study. Although higher *R*^2^ scores were reached when using all bands as predictors than the 145 selected bands (i.e., all except for the PLSR model). In terms of accuracy, higher accuracies (i.e., up to 3 SPAD units below) were reported when using the VI features. Indeed, these results follow what has been suggested in other studies regarding the low (or no) impact of the number of variables in the accuracy estimates, but rather the importance of identifying the marginal effect of the explanatory variables in the dependent variable (Alin, 2010).

Dimensional reduction can be made *via* pruning spectral bands or transforming them into new variables related to plant biochemical traits (as is done herein). (Feilhauer et al., 2015) pruned bands based on regression coefficients (*R*^2^) and metrics that measure band importance, managing to reduce predictors to dozens of Chl absorption channels within the range of 500–750 nm. The same spectral region was fully covered in our study by 15 derivative and continuum-removal based VIs that also inform on Chl content. If we apply this pruning approach to our dataset, it would require approximately 110 bands (Figure 2) to train the models, which is still a large number compared to the available observations (i.e., three samples per plant and 36 plants, for a total of about 108 samples per campaign), leading to the question: what is the impact of not having much larger samples than predictors? A clear example of the impact is evident in the performance of the multivariate linear regression approach presented in this study (“Multiple Model Regression Assessment” and “Multiple Model SPAD Predictions”). When observations do not sufficiently exceed the number of predictors, the least square cost function may overfit the training set, consequently producing poor retrievals. While the other algorithms can cope with this dimensionality issue, a lower-dimensional dataset is desired to improve the computational efficiency of the workflow, and thus feature transformation is a suitable alternative to follow.

Rivera-Caicedo et al. (2017) have investigated dimensionality reduction approaches such as principal components transformation and partial least squares (PLSR), among others. Although some of their trials led to better-fitted models than using all bands, only slight improvements were achieved in terms of accuracy. Similar results were reached in our study when comparing the different ML methods against PLSR (Supplementary Table 2; Figure 6). PLSR reached the best fitting and accuracy scores under the sequential strategy by using band predictors; however, it was exceeded by the random forest and GPR models when using the VIs features under the retraining strategy. Multicollinearity causes this performance by increasing the vulnerability of the predictor weights to vary whenever there is a small change in data, resulting in unstable model performances. Based on the ML algorithm designs (i.e., their mathematical formulations), some are inherently able to handle multicollinearity better than others. For instance, random forest deals well with large dimensional problems due to its pruning strategy, which uses bootstrapping and feature sampling to pick different sets of data and features, and estimate relative importance while training each tree (Supplementary Material; Figure 10B). Alternatively, GPR kernels are coupled with a length scale parameter that measures how strong is each predictor variable in a model (Supplementary Material; Figure 10C). It was possible to perform a comparative analysis through such feature engineering methods to account for coincident relevant variables identified under each approach (Figure 10A). However, although few VIs and spectral ranges were classified as relevant by all three methods, results could still be affected by multicollinearity, especially when features reach similar scores, leading to difficulties in ranking their importance. Developing reduction and transformation dimensionality approaches for hyperspectral data remains a challenge (Thenkabail and Lyon, 2012). As such, new ways to construct alternative prediction variables require continued investigation.

VIs can be considered as a transformed version of the spectral bands that involve known relationships between spectral response and biophysical traits, and hence, are suited to track temporal dynamics along the phenological growth stages. At first glance, such patterns are traceable in our results. For instance, evident Chl dynamics were retrieved by retraining random forest and GPR squared exponential coupled either with VIs or spectral bands under the same strategy (Figure 7B). Such agreement among methods reveals that VIs can be used alternatively as predictor variables. Even more, an exhaustive comparative analysis on the importance of the predictors (“Important Features”) allowed us to trace the spectral information in the most relevant bands and indices (Supplementary Tables 3, 4), revealing that although most of the assessed indices contain the same spectral information covered by the relevant bands, VIs transform the spectral data into new explanatory variables. As such, the VIs approach should be considered as a feature transformation strategy more than a dimensionality reduction method, with the advantages of providing interpretable results and being straightforward to implement in production.

### Which ML Model to Use for Multi-Temporal Retrieving Leaf Chl?

Considering the wide gamut of non-parametric ML methods, the present study sought to examine the most commonly used types of supervised algorithms (Verrelst et al., 2019). Beyond indicating which, if any, particular method could be identified as being the best in estimating Chl metrics from high spatial, spectral and temporal data, some findings can be highlighted based on this study’s data characteristics, the learning strategies, and the subsequent results.

One of the explored methods was support vector regression (SVR) using three different types of kernels and scales (Supplementary Material). Our results showed that SVR linear was the best performing kernel when using the 272 reflectance bands as predictors, although retrieving poorer results when the number of predictors was reduced to 60 VIs. Such behavior suggests that SVR algorithms require a preliminary kernel and feature engineering to fit the regression relationship. In a previous study, (Malenovský et al., 2017) found that the Gaussian SVR outperformed random forest in retrieving total chlorophyll (*C*_ab_) for Antarctic moss by using uni-temporal UAV-based hyperspectral data at a sub-decimeter resolution, training with continuum-removed bands as predictors, and advising to optimize feature selection if intending to use fewer predictors.

Another approach explored in this study was the ensemble of trees. Two general algorithms, boosted and bagged, were tested by training multiple individual medium trees (i.e., ntrees = 60). Specifically, the random forest was explored from the bagged approach, which in general outperformed the boosted ensemble, reaching higher accuracies and better-fitted regressions (Figure 6). However, some of these differences were minor: for instance, when using VI predictors under sequential learning, and for the first and fourth stages under retraining learning, which suggest both methods are suitable for modeling and retrieving multi-temporal Chl content dynamics. The choice of random forest over boosted trees was based on ease of use, since it relies on less tuning parameters than boosted ensembles, and is less prone to overfit when training highly variable or noisy data (Breiman, 2001). It is advised to perform a comparative analysis by training both algorithms, carefully tuning the shrinkage or learning rate parameter in boosting trees, which is decisive in its performance. Similar results have been reported in studies that followed a sequential learning strategy (Shah et al., 2019), finding slightly improved accuracies (i.e., from 5.5 to 3.5 μg/cm^2^ in the RMSE) and better-fitted models (i.e., up to 0.89–0.95 units in the *R*^2^) when training random forest with VIs than when using spectral bands. However, random forest retrievals and validation distributions did best with the retraining learning routine.

Finally, we also examined one of the most promising approaches in hyperspectral remote sensing data analysis: a Bayesian kernel-based method referred to as Gaussian process regression (GPR; Camps-Valls et al., 2016). Four different kernels or covariance functions were compared: exponential, squared exponential (SE), Matern, and rational quadratic, with all of them integrated with a maximum likelihood technique for auto-tuning their parameters. Any of these covariance functions captures the similarity between pairs of observations under the assumption that if the input predictors are close to each other, it is expected that their SPAD values will also be close. Although marginal differences were found among the tested formulations in terms of accuracy with MAE and RMSE (<2 SPAD units), the SE kernel stood out from the rest (Figure 6). Moreover, the assessment metrics for the SE kernel outperformed most of the models examined in this study: only barely surpassed by random forest in some trials using VI predictors and retraining learning. However, SE estimates were associated with high uncertainties when using VIs under sequential learning (~10–15 SPAD units), and for the last two stages when using band features under the retraining routine (~10–12 SPAD units). Accordingly, distributions of the same trials showed discrepancies between the estimates and actual SPAD values from the validation samples, indicating that the GPR model should be subject to optimization routines (Verrelst et al., 2016; Rivera-Caicedo et al., 2017), despite its flexibility and robustness to deal with multi-temporal hyperspectral data.

Few studies in the vegetation spectroscopy literature have extensively compared GPR with other non-parametric regressions (Ashourloo et al., 2016). In two notable examples, (Verrelst et al., 2012); (Rivera-Caicedo et al., 2014) found SE kernel performance exceeded decision trees, neural networks, support vector regression, and kernel ridge methods. To date, most of the ML implementations in hyperspectral applications have tended toward random forest implementations to retrieve biophysical variables (Shah et al., 2019; Vanbrabant et al., 2019). Based on this previous research and the analysis above, the GPR squared exponential, PLSR and random forest were moved to production in the last stage of our workflow, in order to plot the multi-temporal SPAD maps at a pixel level, and including the uncertainty maps produced by GPR, which provides an additional level of information regarding the quality of the estimates. Further analysis based on deep learning and neural networks is recommended to compare their performance under the same dataset.

### Practical Considerations on the Learning Workflow for Chl Retrieval

In general, any non-linear ML approach can be implemented to model and retrieve multi-temporal physiological traits such as Chl content or SPAD levels. However, different strategies and techniques should be explored in order to ensure the efficiency, accuracy, and transferability of model selections. The first general task to pursue in a ML framework should include an exploratory comparative assessment of different models within the training workflow, using the full training/testing data, but predicting over a validation subset. Numerous learning libraries and toolboxes (Rivera-Caicedo et al., 2014) are available for both open-source and commercial applications, including multiple regression algorithms that can be easily implemented to run a preliminary comparative analysis as soon as the first collection of data is available. Selecting the methods to further explore should rely on the data characteristics, such as spatial, spectral, and temporal resolution, and the number of ground-truth samples available.

For chlorophyll monitoring applications specifically, non-destructive *in-situ* sampling can be conducted using chlorophyll meters that provide a relative indicator of leaf Chl content (i.e., SPAD), which can be considered the dependent variable to estimate. However, if the formulations to translate relative units to physical units (i.e., μmol/m^2^) are available, it is advised to translate the data and use Chl content as the dependent variable to estimate (Parry et al., 2014). It is also preferable to use field spectra data to train and fit the models, assuring its comparability with the UAV hyperspectral imagery, which must be radiometrically calibrated and processed in advance (Angel et al., 2020). For structurally vertical, complex, and mixed-species, e.g., orchards or cereals, it is also essential to include BDRF corrections (Aasen and Bolten, 2018). If field spectra data are not available, it may potentially be replaced by synthetic spectral datasets generated through inverting radiative transfer models, although field data is necessary for validation (Feilhauer et al., 2015).

A final consideration relies on quality assessment tasks. Ensuring sufficient observations to split between training, testing, and validation will improve the learning routines and the assessment and model selection stages. In particular, validation is a decisive phase in the ML workflow, and it can be performed by cross-validation when few data are available. When multi-temporal data is involved, poor results can be overlooked if evaluating the validation dataset across time is skipped. Since phenotypic data is dynamic, modeling should be treated as a continuous process by periodically retraining and validating the models, particularly if the new incoming data distribution varies significantly from the first dataset. Such a phenomenon is known as model drift in machine learning literature (Webb et al., 2016) and has to be continuously assessed through different metrics. Complementary to analyzing quality metrics (i.e., RMSE, MAE, *R*^2^), some algorithms like GPR can provide the uncertainty associated with each prediction, allowing uncertainties to be mapped together with model estimates. Accounting for uncertainties associated with SPAD retrieval is of particular interest, since small variations in SPAD units will lead to exponential variations in the actual Chl content (Parry et al., 2014). Multiple evaluation techniques can be further explored to assess different metrics and make improvements accordingly until accurate and coherent results are obtained.

## Conclusions

An innovative machine learning retrieval framework for mapping leaf chlorophyll content across a crop cycle was developed using ultra-high-resolution UAV-based hyperspectral imagery, *in-situ* SPAD observations, and field-based leaf spectra. The workflow evaluates a range of model scenarios to determine the best-performing methods based on the production of accurate and coherent multi-temporal retrievals. The intercomparison of six different ML approaches, including some variations and kernel formulations, accounted for a total of 17 different models. *In-situ* observations were split into a training/testing subset used to fit the models through cross-validation and estimate *R*^2^, and a validation subset was employed to assess the accuracy of the models *via* RMSE and MAE. Three main aspects can be highlighted as innovations from the proposed framework, which match the particular research objectives explored in this study:

- Strategies for selecting the best-suited training/retrieval combinations based on accuracy assessment.
- Evaluation of sequential versus retraining learning strategies.
- Comparison of VIs and spectral band predictors in explaining the SPAD variable.

It was determined that a retraining learning strategy, whereby a model is updated as new data becomes available, proved superior in capturing the temporal dynamics of SPAD-based Chl. In contrast, if models are trained using the full data series at any instance in time (i.e., a sequential learning strategy), only a few model combinations could yield results close to the validation data, vanishing Chl-level changes over time. It was determined that the best combination of training conditions was achieved by coupling sequential learning with spectral bands, and retraining learning with VI predictors. However, given the link that VIs establish between plant traits and spectral responses along the phenology stages, VI predictors may be preferred over spectral bands in order to add interpretability to the models without deteriorating their performance or accuracy. In this direction, PLSR, GPR and random forest were selected as the most promising approaches to optimize and estimate the SPAD predictions. Overall, PLSR and GPR squared exponential outperformed the other models in terms of accuracy and goodness-of-fit when operated under the sequential and retraining strategies, respectively. However, random forest estimates were closer than GPR to the actual validation data distribution, which was used as a reference to evaluate the multi-temporal coherence of the results. An additional assessment element is provided by uncertainty metrics that are included as part of the GPR results. Filtering of the most relevant predictors (bands and VIs) resulted from the inherent feature importance mechanisms of the PLSR, random forest, and GPR approaches. By scoring and classifying the predictors, the selected models reached some agreement on strong individual bands and VIs that highlighted a few decisive spectral ranges and indices useful for retrieving Chl levels.

While a comprehensive assessment of factors contributing to model accuracy and performance was evaluated herein, there remain several further opportunities to advance upon the evaluated approaches, considering the wide range of learning strategies, optimization, and assessment techniques available in open source and commercial applications. Of particular note, there is a need to develop approaches capable of capturing non-evident relationships within large, high-spectral, −temporal, and -spatial datasets to cover canopy scales, and solving prediction problems even under limited *in-situ* training data. Hybrid machine learning with radiative transfer models is a further option to explore the particular leaf and canopy optical properties of agricultural species, integrating other information from UAV systems (e.g., lidar-based canopy height), allowing to account for biochemical and structural traits simultaneously. As data-collection technology evolves, producing ever-larger volumes of data, identifying how best to retrieve accurate informatics quickly and efficiently is an area of critical and much needed research interest. If such techniques and approaches are not developed, we risk being overwhelmed by information, thereby losing the capacity for process insight and knowledge advancement.

## Data Availability Statement

The raw data supporting the conclusions of this article will be made available by the authors, without undue reservation.

## Author Contributions

YA: conceptualization, methodology, software, formal analysis, investigation, visualization, and writing original draft. MFM: conceptualization, methodology, validation, writing review and editing, and supervision. All authors contributed to the article and approved the submitted version.

## Funding

This research was funded by the King Abdullah University of Science and Technology (KAUST).

## Conflict of Interest

The authors declare that the research was conducted in the absence of any commercial or financial relationships that could be construed as a potential conflict of interest.

## Publisher’s Note

All claims expressed in this article are solely those of the authors and do not necessarily represent those of their affiliated organizations, or those of the publisher, the editors and the reviewers. Any product that may be evaluated in this article, or claim that may be made by its manufacturer, is not guaranteed or endorsed by the publisher.
