# Supplementary material for: Machine Learning Strategies for the Retrieval of Leaf-Chlorophyll Dynamics: Model Choice, Sequential Versus Retraining Learning, and Hyperspectral Predictors
Source: Front Plant Sci. 2022 Mar 11;13:722442. doi: 10.3389/fpls.2022.722442 (PMC8963469; doi:10.3389/fpls.2022.722442)
Supplement: Supplementary file 1 [file Data_Sheet_1.docx]

# Supplementary Material Reference

1. Birth et al., 1968 G. Birth, G. McVey, **Measuring the Color of Growing Turf with a Reflectance Spectrophotometer**, Agronomy Journal 60 (1968), pp. 640-643, 10.2134/agronj1968.00021962006000060016x
2. Boegh et al., 2002 E. Boegh, H. Soegaard, N. Broge, C. Hasager, N. Jensen, K. Schelde, A. Thomsen, **Airborne Multi-spectral Data for Quantifying Leaf Area Index, Nitrogen Concentration and Photosynthetic Efficiency in Agriculture**, Remote Sensing of Environment 81 (2002), pp. 179-193, 10.1016/S0034-4257(01)00342-X
3. Boochs et al., 1990 F. Boochs, G. Kupfer, K. Dockter, W. Kühbauch, **Shape of the red edge as vitality indicator for plants**, International Journal of Remote Sensing 11 (1990), pp. 1741-1753, 10.1080/01431169008955127
4. Broge et al., 2000 N. Broge, E. Leblanc, **Comparing Prediction Power and Stability of Broadband and Hyperspectral Vegetation Indices for Estimation of Green Leaf Area and Canopy Chlorophyll Density**, Remote Sensing of Environment 76 (2000), pp. 156-172, 10.1016/S0034-4257(00)00197-8
5. Dash et al., 2007 J. Dash, P.J. Curran, **Evaluation of the MERIS terrestrial chlorophyll index (MTCI)**, Advances in Space Research 39 (2007), pp. 100–104, 10.1016/j.asr.2006.02.034
6. Datt, 1999 B. Datt, **Visible/near infrared reflectance and chlorophyll content in Eucalyptus leaves**, International Journal of Remote Sensing 20 (1999), pp. 2741-2759, 10.1080/014311699211778
7. Daughtry et al., 2000 C.S.T Daughtry, C.L. Walthall, M.S. Kim, E.Brown de Colstoun, J.E. McMurtrey, **Estimating Corn Leaf Chlorophyll Concentration from Leaf and Canopy Reflectance**, Remote Sensing of Environment 74 (2000), pp. 229-239, 10.1016/S0034-4257(00)00113-9
8. Dawson et al., 1998 T.P. Dawson, P.J. Curran, **Technical note A new technique for interpolating the reflectance red edge position**, International Journal of Remote Sensing 19 (1998), pp. 2133-2139, 10.1080/014311698214910
9. Elvidge et al., 1995 C.D. Elvidge, Z. Chen, **Comparison of broad-band and narrow-band red and near-infrared vegetation indices**, Remote Sensing of Environment 54 (1995), pp. 38-48, 10.1016/0034-4257(95)00132-K
10. Filella et al., 1994 I. Filella, J. Penuelas, **The red edge position and shape as indicators of plant chlorophyll content, biomass and hydric status**, International Journal of Remote Sensing 15 (1994), pp. 1459-1470, 10.1080/01431169408954177
11. Filella et al., 1995 I. Filella, L. Serrano, J. Serra, J. Peñuelas, **Evaluating Wheat Nitrogen Status with Canopy Reflectance Indices and Discriminant Analysis**, Crop Science 35 (1995), pp. 1400-1405, 10.2135/cropsci1995.0011183X003500050023x
12. Gamon et al., 1997 J. Gamon, L. Serrano, J. Surfus, **The Photochemical Reflectance Index: An Optical Indicator of Photosynthetic Radiation Use Efficiency Across Species, Functional Types and Nutrient Levels**, Oecologia 112 (1997), pp. 492-501, 10.1007/s004420050337
13. Gamon et al., 1999 J. Gamon, J. Surfus. **Assessing Leaf Pigment Content and Activity with a Reflectometer**, New Phytologist 143 (1999), pp. 105-117, 10.1046/j.1469-8137.1999.00424.x
14. Gitelson et al., 1994 A. Gitelson, M. Merzlyak, **Spectral Reflectance Changes Associated with Autumn Senescence of Aesculus Hippocastanum L. and Acer Platanoides L. Leaves**, Journal of Plant Physiology 143 (1994), pp. 286‑292, 10.1016/S0176-1617(11)81633-0
15. Gitelson et al., 1998 A.A. Gitelson, M. Merzlyak, **Remote Sensing of Chlorophyll Concentration in Higher Plant Leaves**, Advances in Space Research 22 (1998), pp. 689-692, 10.1016/S0273-1177(97)01133-2
16. Gitelson et al. 2001 A.A. Gitelson, A., M. Merzlyak, O.B. Chivkunova, **Optical Properties and Nondestructive Estimation of Anthocyanin Content in Plant Leaves**, Photochemistry and Photobiology 71 (2001), pp. 38-45, 10.1562/0031-8655(2001)0740038OPANEO2.0.CO2
17. Gitelson et al., 2002a A. A. Gitelson, R. Stark, U. Grits, D. Rundquist, Y. Kaufman, D. Derry, **Vegetation and Soil Lines in Visible Spectral Space: A Concept and Technique for Remote Estimation of Vegetation Fraction**, International Journal of Remote Sensing 23 (2002), pp. 2537−2562, 10.1080/01431160110107806
18. Gitelson et al. 2002b A.A. Gitelson, Y. Zur, O.B. Chivkunova, M.N. Merzlyak, **Assessing Carotenoid Content in Plant Leaves with Reflectance Spectroscopy**, Photochemistry and Photobiology 75 (2002), pp. 272-281, 10.1562/0031-8655(2002)0750272ACCIPL2.0.CO2
19. Gitelson et al., 2003 A.A. Gitelson, A. Viña, T.J. Arkebauer, D.C. Rundquist, G. Keydan, B. Leavitt, **Remote estimation of leaf area index and green leaf biomass in maize canopies**, Geophys. Res. Lett. 30 (2003), pp. 1248, 10.1029/2002GL016450
20. Goel et al., 1994 N. Goel, W. Qin, **Influences of Canopy Architecture on Relationships between Various Vegetation Indices and LAI and Fpar: A Computer Simulation**, Remote Sensing Reviews 10 (1994), pp. 309-347, 10.1080/02757259409532252
21. Guyot et al., 1998 G. Guyot, F. Baret, **Utilisation de la haute résolution spectrale pour suivre l'état des couverts végétaux**, Proceedings of the 4th International colloquium on spectral signatures of objects in remote sensing, ESA SP-287, Assois, France (1988), pp. 279-286
22. Haboudane et al., 2004 D. Haboudane, J.R. Miller, E. Pattey, P.J. Zarco-Tejada, I.B. Strachan, **Hyperspectral Vegetation Indices and Novel Algorithms for Predicting Green LAI of Crop Canopies: Modeling and Validation in the Context of Precision Agriculture**, Remote Sensing of Environment 90 (2004), pp. 337-352, 10.1016/j.rse.2003.12.013
23. Huete et al., 2002 A. Huete, K. Didan, T. Miura, E.P. Rodriguez, X. Gao, L.G. Ferreira, **Overview of the Radiometric and Biophysical Performance of the MODIS Vegetation Indices**, Remote Sensing of Environment 83 (2002), pp. 195–213, 10.1016/S0034-4257(02)00096-2
24. Huete, 1988 A.R. Huete, **A Soil-Adjusted Vegetation Index (SAVI)**, Remote Sensing of Environment 25 (1988), pp. 295-309, 10.1016/0034-4257(88)90106-X
25. Kaufman et al., 1992 Y. Kaufman, D. Tanre, **Atmospherically Resistant Vegetation Index (ARVI) for EOS-MODIS**, IEEE Transactions on Geoscience and Remote Sensing 30 (1992), pp. 261-270, 10.1109/36.134076
26. Kokaly et al., 1999 R.F. Kokaly, R.N. Clark, **Spectroscopic Determination of Leaf Biochemistry Using Band-Depth Analysis of Absorption Features and Stepwise Multiple Linear Regression**, Remote Sensing of Environment 67 (1999), pp. 267-287, 10.1016/S0034-4257(98)00084-4
27. Lobell et al., 2003 D. Lobell, G. Asner, **Hyperion studies of crop stress in Mexico**, Proceedings of the 12th Annual JPL Airborne Earth Science Workshop, Pasadena, CA, USA, 24–28 February 2003
28. Main et al., 2011 R. Main, M. A. Cho, R. Mathieu, M.M. O’Kennedy, A. Ramoelo, S. Koch, **An investigation into robust spectral indices for leaf chlorophyll estimation**, ISPRS Journal of Photogrammetry and Remote Sensing 66 (2011), pp. 751-761, 10.1016/j.isprsjprs.2011.08.001
29. Malenovský et al., 2006 Z. Malenovský, C. Ufer, Z. Lhotáková, J.P.G.W. Clevers, M.E. Schaepman, J. Albrechtová, P. Cudlín, **A new hyperspectral index for chlorophyll estimation of a forest canopy: area under curve normalized to maximal band depth between 650–725 nm**, EARSeL eProceedings5 (2006), pp. 161–172, 10.5167/uzh-62112
30. Merzlyak et al., 1999 J. Merzlyak, A.A. Gitelson, O.B. Chivkunova, V.Y. Rakitin, **Non-destructive Optical Detection of Pigment Changes During Leaf Senescence and Fruit Ripening**, Physiologia Plantarum 106 (1999), pp. 135-141, 10.1034/j.1399-3054.1999.106119.x
31. Penuelas et al., 1993 J. Peñuelas, I. Filella, C. Biel, l. Serrano, R. Savé, **The Reflectance at the 950-970 Region as an Indicator of Plant Water Status**, International Journal of Remote Sensing 14 (1993), pp.1887-1905, 10.1080/01431169308954010
32. Peñuelas et al., 1994 J. Peñuelas, J.A. Gamon, A.L. Fredeen, J. Merino, C.B. Field**, Reflectance indices associated with physiological changes in nitrogen- and water-limited sunflower leaves**, Remote Sensing of Environment 48 (1994), pp. 135-146, 10.1016/0034-4257(94)90136-8
33. Penuelas et al., 1995 J. Penuelas, J., F. Baret, I. Filella, **Semi-Empirical Indices to Assess Carotenoids/Chlorophyll-a Ratio from Leaf Spectral Reflectance**, Photosynthetica 31 (1995), pp. 221-230
34. Rondeaux et al., 1996 G. Rondeaux, M. Steven, F. Baret, **Optimization of Soil-Adjusted Vegetation Indices**, Remote Sensing of Environment 55 (1996), pp. 95-107, 10.1016/0034-4257(95)00186-7
35. Rouse et al., 1917 J.W. Jr. Rouse, R.H. Haas, J. Schell, D. Deering, **Monitoring Vegetation Systems in the Great Plains with ERTS**, Third ERTS Symposium, NASA (1973), pp. 309-317
36. Sanches et al., 2014 I.D.A. Sanches, C.R.S. Filho, R.F. Kokaly, **Spectroscopic remote sensing of plant stress at leaf and canopy levels using the chlorophyll 680nm absorption feature with continuum removal**, ISPRS Journal of Photogrammetry and Remote Sensing 97 (2014), pp. 111-122, 10.1016/j.isprsjprs.2014.08.015
37. Sims et al., 2002 D. Sims, J. Gamon, **Relationships Between Leaf Pigment Content and Spectral Reflectance Across a Wide Range of Species, Leaf Structures and Developmental Stages**, Remote Sensing of Environment 81 (2002), pp. 337-354, 10.1016/S0034-4257(02)00010-X
38. Sripada et al., 2006 R.P. Sripada, R.W. Heiniger, J.G White, A.D. Meijer, **Aerial Color Infrared Photography for Determining Early In-season Nitrogen Requirements in Corn**, Agronomy Journal 98 (2006), pp. 968-977, 10.2134/agronj2005.0200
39. Vogelmann et al., 1993 J. Vogelmann, B. Rock, D. Moss, **Red Edge Spectral Measurements from Sugar Maple Leaves**, International Journal of Remote Sensing 14 (1993), pp. 1563-1575, 10.1080/01431169308953986
40. Wu et al., 2008 C. Wu, Z. Niu, Q. Tang, W. Huang, **Estimating chlorophyll content from hyperspectral vegetation indices: Modeling and validation**, Agricultural and Forest Meteorology 148 (2008), pp. 1230-1241, 10.1016/j.agrformet.2008.03.005
41. Zhao et al., 2014 J. Zhao, M. Feng, C. Wang, W. Yang, Z. Li, Z. Zhu, P. Ren, T. Liu, H. Wang, **Simulating the Content of Chlorophyll in Winter Wheat Based on Spectral Vegetation Index**, Journal of Shanxi Agricultural University (Natural Science Edition) 34 (2014), pp. 391–396
