# Supplementary material for: Machine Learning Strategies for the Retrieval of Leaf-Chlorophyll Dynamics: Model Choice, Sequential Versus Retraining Learning, and Hyperspectral Predictors
Source: Front Plant Sci. 2022 Mar 11;13:722442. doi: 10.3389/fpls.2022.722442 (PMC8963469; doi:10.3389/fpls.2022.722442)
Supplement: Supplementary file 2 [file Data_Sheet_2.docx]

Supplementary Material

# Supplementary Methods

Following is a brief overview of the chosen regression methods, including multivariate linear, partial-least-square, decision trees, ensemble trees, support vector regression, and Gaussian process, with further details provided in the associated references.

**Multivariate Linear regression**

Multivariate linear regression attempts to establish a relationship between the dependent variable$y_{i}$ and one or more independent variables or predictors $x_{j}$ (1), by fitting a linear prediction equation (2) to observed data, using the method of least squares (3) (Anderson, 2003).

$y_{i}= f\left(x_{i}\right)+ \varepsilon_{i} =\beta_{0}+ \beta_{1}x_{i1}+ \beta_{2}x_{i2}+\cdots+\beta_{j}x_{ij}+ \varepsilon_{i}, i=1, 2,\cdots,n j=1, 2,\cdots, m$ (1)

$\hat{y}_{i}= \beta_{0}+ \beta_{1}x_{i1}+ \beta_{2}x_{i2}+\cdots+\beta_{j}x_{ij}$ (2)

$\min\sum_{i =1}^{n} \left(y_{i}-\hat{y}_{i}\right)^{2}$ (3)

where $n$is the number of observations, $m$ is the number of predictors, $\beta_{0}$ is the intercept term, $\beta_{j}$ coefficients express the impact of predictors $x_{j}$ on the mean of the response$y_{i}$, $x_{ij}$ is the $i$th observation on the $j$th predictor, and $\varepsilon_{i}$ are noise terms normally distributed with mean zero and constant variance *σ^2^*. The model (1) is fitted by finding the values of *β_0_* and *β_j_* that minimize the sum of the squared differences between $y_{i}$ and the predictions $\hat{y}_{i}$ (2).

**Partial Least-Squares regression (PLSR)**

Partial least squares is a method that models the relationships between the dependent and independent sets of variables by projecting them into a low-dimensional subspace of components or latent variables (4). Such components are retrieved by maximizing the covariance between features *x* and response *y* and maximizing their individual variances. The score components serve as new predictors to regress *y* and inspect how strongly each component depends on the original independent variables *x* (5). Based on the weights (6), PLSR can be used for dimensionality reduction tasks by retaining only the *x* variables contributing the most to each component (Rosipal et al., 2006) and comparing the relevance of the predictors with other models through using the weights as an index.

$Y= X\beta+ \varepsilon$ (4)

$Y= T_{h}C_{h}^{'}+ \varepsilon_{h} =XW_{h}C_{h}^{'}+\varepsilon_{h}=XW_{h}(P_{h}^{'}W_{h})^{-1}C_{h}^{'}+\varepsilon_{h}$ (5)

$\beta=W_{h}\left(P_{h}^{'}W_{h}\right)^{-1}C_{h}^{'}$ (6)

where $Y$is the matrix of the dependent variable, $X$is the matrix of the explanatory variables, $T$is the matrix of *x*-scores,$C$ is the *y*-loading matrix, $P$ is the x-loading matrix, $W$is the x-weight matrix, and $\varepsilon$is the matrix of residuals. The regression coefficients of $Y$on $X$are comprised in the matrix $\beta$, with $h$components retrieved by PLSR.

**Individual decision trees**

Binary tree-based models establish non-linear relationships between the response $y_{i}$ and the predictors $x_{j}$ by flowing data down from a root node to leaves (7) (8) (Kamiński et al., 2018). Training data is sequentially split into smaller samples according to specific cutoff conditional values of the predictor variables, creating different intermediate leaf nodes. Once the terminal splits are reached, the average outcome of the training data contained in each end leaf node is used to retrieve a prediction. It is essential to consider that simple trees prevent overfitting, but their predictive power could be compromised; in contrast, leafy trees are usually highly accurate but tend to overfit. An option to balance these aspects is by optimizing the minimum leaf size, which is a limit to stop splitting nodes once the number of observations in an intermediate node reaches the defined minimum. In this study, three types of decision trees are evaluated based on this parameter: fine, medium, and coarse. As a rule of thumb, the optimization should start with a fine tree whose minimum leaf size is 5, yielding a leafy tree with many small leaves. Then, a medium tree with a minimum leaf size of 10. Finally, a coarse tree of a few large leaves with a minimum size of 20.

$\hat{y}_{i}= \sum_{k=1}^{l} a_{k}I , k=1, 2, \cdots, l$ (7)

$I= \left\{\begin{matrix} 1, if x_{i}\in S_{k} \\ 0, otherwise \end{matrix}\right. , i=1, 2, \cdots, n$ (8)

When predicting $n$ observations using a decision tree of $l$ leaf nodes, each observation $x_{i}$ falls into a single end leaf node$S_{k}$. The algorithm returns an identical function $I$ of ones if the observation $x_{i}$ is in the subset$S_{k}$, or zero otherwise. Thus, the prediction $\hat{y}_{i}$ is the average of all training samples $a_{k}$ in$S_{k}$.

**Ensembles of decision trees**

The response $y_{i}$ is also determined by using two widely known methods of ensembles of decision trees: bagging and boosting. Under the bagging model, decision trees are grown simultaneously from multiple replicas of the training dataset and randomly sampled with replacement. The number of learned trees is set to 60, following the tuning procedure of previous research using similar datasets (see Shah et al., 2019 for further details), suggesting that the optimal amount of trees can vary between 60 and 120 with no significant performance gain (Oshiro et al., 2012; Breiman, 2001). Splitting operates under an interaction-detection technique (Loh, 2002) designed to reduce issues caused by bias in variable selection when predictors and response are highly inter-correlated, thus retrieving better predictor importance estimates than standard techniques that rely on a defined number of input variables. From a bag that contains one-third of the total number of predictors $x_{j}$, the interaction-detection runs two chi-square tests of independence: one between each pair of predictors and the response variable, and the other between each predictor and the response. Branch nodes are split level by level using the predictor that minimizes the p-value (< 0.05) of both tests until all tests can no longer be minimized ( p-value > 0.05). Then, the predicted response $\hat{y}_{i}$ is estimated by averaging individual predictions from each tree. The importance of each predictor is estimated through a permutation accuracy metric during the fitting process. For each tree *T* in the forest, the prediction error is calculated on the out-of-bag (OOB) training data; then, the same calculation is performed by randomly permuting the values of each predictor. Finally, the difference in prediction errors are averaged over all the forest for each variable. Thus, the worse a model performs when permuting a variable, the more relevant that variable is in the model.

Alternatively, under the boosting approach, trees are learned sequentially by fitting each new tree to the difference between the sampled response $y_{i}$ and the accumulated prediction of all trees previously grown, minimizing the mean squared error (Dietterich, 2000b). The leaf size is set to 10 for both approaches to compare with the individual medium decision tree performance.

**Support vector regression**

Support vector regression (SVR) performs multivariable linear regression in a higher-dimensional space (Awad et al., 2015). Assuming $f\left(x\right)$ (9) is a straight line going through the$y$ axis, the objective of the SVR algorithm is to find a flattened (10) hyperplane or regression function$f\left(x\right)$ with the widest buffer zone that contains the maximum number of training data points$y_{i}$. This margin of tolerance defines a decision boundary at an $\varepsilon$ distance from either side of the hyperplane (11), and observations$y_{i}$ on the edge and within the decision boundary swath, are called support vectors.

$f\left(\mathbf{x}\right)=\mathbf{\beta}\cdot\mathbf{x}+b$ (9)

$\min\frac{1}{2}\left\|\mathbf{\beta}^{2}\right\|$ (10)

$subject to \left\{\begin{matrix} y_{i}-\mathbf{\beta}\cdot\mathbf{x}_{\mathbf{i}}-b \leq\varepsilon\\ \mathbf{\beta}\cdot\mathbf{x}_{\mathbf{i}}+b-y_{i} \leq\varepsilon\end{matrix}\right.$ (11)

This optimization problem is solved by constructing a Lagrange function with dual positive multipliers $\alpha_{i}$ and $\alpha_{i}^{*}$ for each training observation $\mathbf{x}_{\mathbf{i}}$, which leads to a linear combination to describe $\mathbf{\beta}$ (12), and a fitted function (13) for new predictions.

$\mathbf{\beta}= \sum_{i=1}^{n} \left(\alpha_{i}-\alpha_{i}^{*}\right)\mathbf{x}_{\mathbf{i}}$ (12)

$f\left(\mathbf{x}\right)= \sum_{i=1}^{n} \left(\alpha_{i}-\alpha_{i}^{*}\right)K\left(\mathbf{x}_{\mathbf{i}}\cdot\mathbf{x}\right)+b$ (13)

In this study, we explore additional non-linear SVR cases, replacing the linear kernel$K_{l}$ (14) by using a polynomial kernel $K_{q}$ of order $q=2, 3$(15) and a Gaussian kernel $K_{G}$, also known as a radial basis function (RBF) in the literature (16).

$K_{l}\left(\mathbf{x}_{\mathbf{i}},\mathbf{x}\right)=\left(\mathbf{x}_{\mathbf{i}}\cdot\mathbf{x}\right)$ (14)

$K_{q}\left(\mathbf{x}_{\mathbf{i}},\mathbf{x}\right)=\left(1+\mathbf{x}_{\mathbf{i}}\cdot\mathbf{x}\right)^{q}$ (15)

$K_{G}\left(\mathbf{x}_{\mathbf{i}},\mathbf{x}\right)=\exp\left(-\gamma\left\|\mathbf{x}_{\mathbf{i}}-\mathbf{x}\right\|^{2}\right)$ (16)

where $\gamma$is a positive scale parameter that sets the width of the Gaussian curve.

**Gaussian processes**

Gaussian process regression (GPR) is a kernel-based probabilistic approach that not only fits one regression function, but a family of non-linear multivariate functions over the set of observed predictors (Rasmussen et al., 2005). Unlike other machine learning methods that only calculate a prediction value for each observation, GPR also retrieves confidence intervals of the prediction. Assuming the linear function (1), where$\mathbf{x}$ is the input vector of predictors, and$y$ is the scalar output of the dependent variable,$f\left(\mathbf{x}\right)$ is considered as a Gaussian process whose prior has a zero mean and covariance$\mathbf{k}= k\left(\mathbf{x},\mathbf{x}'\right)$. Thus, a new prediction $y_{*}$ given an observation $\mathbf{x}_{*}$ can be estimated by a joint distribution (20) over the training and testing datasets.

(17)

(18)

where$\mathbf{A}=\mathbf{k}+\sigma^{2}\mathbf{I}$, $\mathbf{I}$ is an $n \times n$ identity matrix, $\mathbf{k}_{*}= \left[k\left(\mathbf{x}_{*,}\mathbf{x}_{1}\right), \cdots, k\left(\mathbf{x}_{*,}\mathbf{x}_{n}\right)\right]^{T}$ and$\text{A}_{*}=k\left(\mathbf{x}_{*},\mathbf{x}_{*}\right)+\sigma^{2}$. The prediction $\text{y}_{*}$ follows a normal distribution and is expressed in terms of a mean $\text{μ}_{*}$ (19), and the uncertainty of the prediction $\text{σ}_{*}^{2}$ (20).

$\mu_{*}\left(\mathbf{x}_{*}\right)= \mathbf{k}_{*}^{T}\left(\mathbf{k}+ \sigma^{2}\mathbf{I}\right)^{-1}\mathbf{y}$ (19)

$\text{σ}_{*}^{2}\left(\mathbf{x}_{*}\right)= \text{A}_{*}-\mathbf{k}_{*}^{T}\left(\mathbf{k}+\sigma^{2}\mathbf{I}\right)^{-1}\mathbf{k}_{*}$ (20)

The covariance matrix $\mathbf{k}$ is parameterized by a kernel function that measures the similarity between predictor values$\mathbf{x}$ in order to retrieve correlated predictions$\mathbf{y}$. In this study, four different covariance functions (or kernels) are evaluated: exponential $\mathbf{k}_{exp}$ (21), squared exponential $\mathbf{k}_{sq}$ or RBF (22), rational quadratic $\mathbf{k}_{rq}$ (23), and Matern $\mathbf{k}_{M}$ (24).

$k_{exp}\left(\mathbf{x}, \mathbf{x}'\text{|}\mathbf{\theta}\right)= \text{υ} exp\left(-r\right)$ (21)

$k_{sq}\left(\mathbf{x}, \mathbf{x}'\text{|}\mathbf{\theta}\right)= \text{υ} exp\left(-\frac{1}{2}r^{2}\right)$ (22)

$k_{rq}\left(\mathbf{x}, \mathbf{x}'\text{|}\mathbf{\theta}\right)= \text{υ}\left(1+ \frac{1}{2\alpha}r^{2}\right)^{-\alpha}$ (23)

$k_{M} \left(\mathbf{x}, \mathbf{x}'\text{|}\mathbf{\theta}\right)= \text{υ} \left(1+ \sqrt{3}r\right)\text{exp}\left(-\sqrt{3}r\right)$ (24)

where $r= \sqrt{\frac{1}{2}\sum_{j=1}^{m} \frac{\left(x_{j}-x_{j}^{'}\right)^{2}}{l_{j}^{2}}}$ for $j=1, 2, \cdots, m$ number of predictors.

The vector of parameters $\mathbf{\theta}= \left[\text{υ}, l_{j}\right]$ parameterizes these kernels, also known as automatic relevance determination (ARD) structure, which is available in some band analysis tools (BAT) (Rivera-Caicedo et al., 2017). $\mathbf{\theta}$ is composed of a smoothing factor $\text{υ}$, and a length scale $\text{l}_{j}$ that informs on the relevance of each predictor in the model through a weakness index.

# Supplementary Figures and Tables

**Supplementary Table 1.** List of Vegetation Indices. The first set contains widely used broadband greenness indices that explore the correlation between the near-infrared peak and the deepest absorption in the red range of the vegetation spectra to account for the gross chlorophyll amount at a canopy level. The second group involves narrowband greenness indices focused on studying the transition between the red absorption and the near-infrared (650 nm - 750 nm), commonly known as the red-edge range, allowing an estimate of leaf and canopy chlorophyll content. The third set of VIs examines the light use efficiency for photosynthesis and leaf redness by measuring the ratio of carotenoids and anthocyanins to chlorophyll, using blue, red, and near-infrared narrowbands, and minimizing leaf structural confounding effects. The fourth group accounts for plant senescence by assessing the carotenoid to chlorophyll ratio. A fifth group of VIs determines stress levels based on leaf pigments, where bands between 510 nm and 550 nm are used to measure carotenoid concentration, and bands around 550 nm and 750 nm are used to evaluate anthocyanin content. A sixth group comprises a leaf water content index was used to consider the indirect relationship between water status and leaf chlorophyll (Penuelas et al., 1993) by measuring a characteristic absorption feature in the range from 900 nm to 970 nm, which is also observed by the SPAD chlorophyll meter (e.g., ~940 nm). The seventh group comprises indices for estimating chlorophyll a and b content based on absorption peaks around 430 nm, 550 nm, 670 nm, 705 nm, and absorption minimums around 750 nm, 780 nm, 800 nm and 860 nm, to quantify the difference between high near-infrared reflectance and red light absorption. A group of derivative VIs was included to investigate the relationship between slope and curvatures of the vegetation spectra with leaf chlorophyll content, with most of these focused on the red edge range and a few on the green peak range. In the final VI grouping, continuum removal-based indices in the range 550 nm to 750 nm were included to account for the close relationship between absorption features, such as the area under the curve and maximum depth, with leaf chlorophyll content.

| **#** | **Name** | **VI** | **Formulation** | **Group** | **Reference** |
| --- | --- | --- | --- | --- | --- |
| 1 | Normalized Difference Vegetation Index | NDVI | $\frac{R_{860}-R_{650}}{R_{860}-R_{650}}$ | Broadband Greenness | Rouse et al., 1917 |
| 2 | Simple Ratio Index | SRI | $\frac{R_{860}}{R_{650}}$ |  | Birth et al., 1968 |
| 3 | Non-Linear Index | NLI | $\frac{R_{880}^{2}-R_{670}}{R_{880}^{2}+R_{670}}$ |  | Goel et al., 1994 |
| 4 | Visible Atmospherically Resistant Index [green] | VARI | $\frac{R_{555}-R_{650}}{R_{555}+R_{650}-R_{475}}$ |  | Gitelson et al., 2002a |
| 5 | Enhanced Vegetation Index | EVI | $\frac{R_{860}-R_{650}}{R_{860}+\left(6\times R_{650}\right)-\left(7,5\times R_{450}\right)+1}$ |  | Huete et al., 2002 |
| 6 | Leaf Area Index | LAI | $\left(3,618\times EVI\right)-0,118$ |  | Boegh et al., 2002 |
| 7 | Atmospherically Resistant Vegetation Index | ARVI | $\frac{R_{860}-\left(R_{650}-\left(R_{470}-R_{650}\right)\right)}{R_{860}+\left(R_{650}-\left(R_{470}-R_{650}\right)\right)}$ | Narrowband Greenness | Kaufman et al., 1992 |
| 8 | Red Edge Normalized Difference Vegetation Index | RENDVI | $\frac{R_{750}-R_{705}}{R_{750}+R_{705}}$ |  | Gitelson et al., 1994 |
| 9 | Modified Red Edge Simple Ratio Index | MRESRI | $\frac{R_{750}-R_{445}}{R_{705}-R_{445}}$ |  | Sims et al., 2002 |
| 10 | Modified Red Edge Normalized Difference Vegetation Index | MRENDVI | $\frac{R_{750}-R_{705}}{R_{750}+R_{705}-\left(2\times R_{445}\right)}$ |  | Sims et al., 2002 |
| 11 | Sum Green Index | SGI | $\frac{\sum_{i=500}^{N=600} R_{i}}{N}$ |  | Lobell et al., 2003 |
| 12 | Vogelmann Red Edge Index 1 | VREI1 | $\frac{R_{740}}{R_{720}}$ |  | Vogelmann et al., 1993 |
| 13 | Vogelmann Red Edge Index 2 | VREI2 | $\frac{R_{734}-R_{747}}{R_{715}+R_{726}}$ |  | Vogelmann et al., 1993 |
| 14 | Red Edge Position Index (4 Point Linear Interpolation) | REPI3 | $700+\left[40\times\left(\frac{\left(\frac{R_{670}+R_{780}}{2}\right)-R_{700}}{R_{740}-R_{700}}\right)\right]$ |  | Guyot et al., 1998 |
| 15 | Red Edge Position Index (Guyot and Baret) | REPI4 | $705+\left[35\times\left(\frac{\left(\frac{R_{665}+R_{783}}{2}\right)-R_{705}}{R_{740}-R_{705}}\right)\right]$ |  | Guyot et al., 1998 |
| 16 | Photochemical Reflectance Index | PRI | $\frac{R_{531}-R_{570}}{R_{531}+R_{570}}$ | Light Use Efficiency | Gamon et al., 1997 |
| 17 | Structure Insensitive Pigment Index | SIPI | $\frac{R_{800}-R_{445}}{R_{800}-R_{680}}$ |  | Penuelas et al., 1995 |
| 18 | Red Green Ratio Index | RGRI | $\frac{\sum_{i=600}^{699} R_{i}}{\sum_{i=500}^{599} R_{i}}$ |  | Gamon et al., 1999 |
| 19 | Plant Senescence Reflectance Index | PSRI | $\frac{R_{680}-R_{500}}{R_{750}}$ | Senescent | Merzlyak et al., 1999 |
| 20 | Carotenoid Reflectance Index 1 | CRI1 | $\frac{1}{R_{510}}-\frac{1}{R_{500}}$ | Leaf Pigments-based Stress | Gitelson et al. 2002b |
| 21 | Carotenoid Reflectance Index 2 | CRI2 | $\frac{1}{R_{510}}-\frac{1}{R_{700}}$ |  | Gitelson et al. 2002b |
| 22 | Anthocyanin Reflectance Index 1 | ARI1 | $\frac{1}{R_{550}}-\frac{1}{R_{700}}$ |  | Gitelson et al. 2001 |
| 23 | Anthocyanin Reflectance Index 2 | ARI2 | $R_{800}\times\left(\frac{1}{R_{550}}-\frac{1}{R_{700}}\right)$ |  | Gitelson et al. 2001 |
| 24 | Water Band Index | WBI | $\frac{R_{900}}{R_{970}}$ | Water Content | Penuelas et al., 1993 |
| 25 | Modified Chlorophyll Absorption Ratio Index | MCARI | $\left(R_{750}-R_{705}\right)-\left[\left(0,2\times\left(R_{750}-R_{550}\right)\right)\times\frac{R_{750}}{R_{705}}\right]$ | Leaf Chl | Daughtry et al., 2000 |
| 26 | Modified Chlorophyll Absorption Ratio Index Improved | MCARI2 | $\frac{1,5\times\left[\left(2,5\times\left(R_{800}-R_{670}\right)\right)-\left(1,3\times\left(R_{800}-R_{550}\right)\right)\right]}{\sqrt{\left(\left(\left(2\times R_{800}\right)+1\right)^{2}\right)-\left(\left(6\times R_{800}\right)-\left(5\times\sqrt{R_{670}}\right)\right)-0,5}}$ |  | Haboudane et al., 2004 |
| 27 | Modified Triangular Vegetation Index | MTVI | $1,2\times\left[\left(1,2\times\left(R_{800}-R_{550}\right)\right)-\left(2,5\times\left(R_{670}-R_{550}\right)\right)\right]$ |  | Haboudane et al., 2004 |
| 28 | Modified Triangular Vegetation Index - Improved | MTVI2 | $\frac{1,5\times\left[\left(1,2\times\left(R_{800}-R_{550}\right)\right)-\left(2,5\times\left(R_{670}-R_{550}\right)\right)\right]}{\sqrt{\left(\left(\left(2\times R_{800}\right)+1\right)^{2}\right)-\left(\left(6\times R_{800}\right)-\left(5\times\sqrt{R_{670}}\right)\right)-0,5}}$ |  | Haboudane et al., 2004 |
| 29 | Soil Adjusted Vegetation Index | SAVI | $\frac{1,5\times\left(R_{750}-R_{705}\right)}{R_{750}-R_{705}}$ |  | Huete, 1988 |
| 30 | Optimized Soil Adjusted Vegetation Index | OSAVI | $\frac{\left(1+0,16\right)\times\left(R_{750}-R_{705}\right)}{R_{750}+R_{705}+0,16}$ |  | Rondeaux et al., 1996 |
| 31 | MCARI/OSAVI | MCARI/OSAVI | $\frac{MCARI}{OSCAVI}$ |  | Wu et al., 2008 |
| 32 | Transformed Chlorophyll Absorption Reflectance Index | TCARI | $3\times\left[\left(R_{750}-R_{705}\right)-\left(0,2\times\left(R_{750}-R_{550}\right)\times\left(\frac{R_{750}}{R_{705}}\right)\right)\right]$ |  | Haboudane et al., 2004 |
| 33 | TCARI/OSAVI | TCARI/OSAVI | $\frac{TCARI}{OSAVI}$ |  | Wu et al., 2008 |
| 34 | Triangular Vegetation Index | TVI | $\left(60\times\left(R_{750}-R_{550}\right)\right)-\left(100\times\left(R_{670}-R_{550}\right)\right)$ |  | Broge et al., 2000 |
| 35 | Green Normalized Difference Vegetation Index | GNDVI | $\frac{R_{860}-R_{550}}{R_{860}+R_{550}}$ |  | Gitelson et al., 1998 |
| 36 | Normalized Chlorophyll Ratio Index | NPCI | $\frac{R_{680}-R_{430}}{R_{680}-R_{430}}$ |  | Peñuelas et al., 1994 |
| 37 | Green Ratio Vegetation Index | GRVI | $\frac{R_{860}}{R_{550}}$ |  | Sripada et al., 2006 |
| 38 | Red Edge Chlorophyll Index | NDCI1 | $\frac{R_{783}}{R_{705}}-1$ |  | Gitelson et al., 2003 |
| 39 | Green Chlorophyll Index | NDCI2 | $\frac{R_{783}}{R_{560}}-1$ |  | Gitelson et al., 2003 |
| 40 | MERIS Terrestrial Chlorophyll Index | MTCI | $\frac{R_{740}-R_{705}}{R_{705}+R_{655}}$ |  | Dash et al., 2007 |
| 41 | Vogelmann Red Edge Derivative Index | VREDI | $\frac{D_{715}}{D_{705}}$ | Derivative Leaf Chl | Vogelmann et al., 1993 |
| 42 | Red Edge Position Index (Max. First Derivative) | REPI1 | $\lambda_{max_{\left(D_{690},D_{740}\right)}}$ |  | Filella et al., 1994 |
| 43 | Derivative of the REP | RE | $max \left(D_{690},D_{740}\right)$ |  | Filella et al., 1994 |
| 44 | Red Edge Position Index (Polynomial Fitting) | REPI2 | $\frac{\left\{-\left\{\left[D_{705}-\left(\frac{705\times\left(D_{676}-D_{705}\right)}{676-705}\right)\right]-\left[D_{762}-\left(\frac{762\times\left(D_{719}-D_{762}\right)}{719-762}\right)\right]\right\}\right\}}{\left(\frac{D_{676}-D_{705}}{676-705}\right)-\left(\frac{D_{719}-D_{762}}{719-762}\right)}$ |  | Dawson et al., 1998 |
| 45 | D703 | D703 | $D_{703}$ |  | Boochs et al., 1990 |
| 46 | D720 | D720 | $D_{720}$ |  | Boochs et al., 1990 |
| 47 | Red Edge and Green Derivative Normalized | EGFN | $\frac{\left(max \left(D_{690},D_{740}\right)\right)-\left(max \left(D_{509},D_{536}\right)\right) }{\left(max \left(D_{690},D_{740}\right)\right)+\left(max \left(D_{509},D_{536}\right)\right)}$ |  | Filella et al., 1994 |
| 48 | Red Edge and Green Derivative Ratio | EGFR | $\frac{\left(max \left(D_{690},D_{740}\right)\right) }{\left(max \left(D_{509},D_{536}\right)\right)}$ |  | Filella et al., 1994 |
| 49 | Sum of First Derivative | Sum1 | $\sum_{i=680}^{780} D_{i}$ |  | Filella et al., 1995 |
| 50 | Derivative Green Vegetation Index using Zero baseline | DZ_DGVI | $\sum_{i=626}^{795} \left[\left\vert D_{i}\right\vert\times\left(\lambda_{i}-626\right)\right]$ |  | Elvidge et al., 1995 |
| 51 | Datt | Datt | $\frac{D_{754}}{D_{704}}$ |  | Datt, 1999 |
| 52 | DPI | DPI | $\frac{D_{688}\times D_{710}}{D_{697}^{2}}$ |  | Main et al., 2011 |
| 53 | Derivative Ratio 1 | DSR1 | $\frac{D_{730}}{D_{706}}$ |  | Main et al., 2011 |
| 54 | Derivative Ratio 2 | DSR2 | $\frac{D_{705}}{D_{722}}$ |  | Main et al., 2011 |
| 55 | FDNDVI | FDNDVI | $\frac{D_{630}-D_{723}}{D_{630}+D_{723}}$ |  | Zhao et al., 2014 |
| 56 | Maximal Band Depth of the Continuum-Removed | MBD | $1-\left[min \left(CR_{555},CR_{752}\right)\right]$ | Continuum-removed Chl | Kokaly et al., 1999 |
| 57 | Area Under Continuum-Removed | AUC | $0,5\times\left(\sum_{i=555}^{752} \left[\left(\lambda_{i+1}-\lambda_{i}\right)\times\left(CR_{i+1}+CR_{i}\right)\right]\right)$ |  | Malenovský et al., 2006 |
| 58 | Area Under Curve Normalized to Maximal Band Depth | ANMB | $\frac{AUC}{MBD}$ |  | Malenovský et al., 2006 |
| 59 | Leaf Plant Stress Detection Index | LPSDI | $\frac{\left(1-CR_{575}\right)-\left(1-MBD\right)}{\left(1-CR_{575}\right)+\left(1-MBD\right)}$ |  | Sanches et al., 2014 |
| 60 | Canopy Plant Stress Detection Index | CPSDI | $\frac{\left(1-CR_{560}\right)-\left(1-MBD\right)}{\left(1-CR_{560}\right)+\left(1-MBD\right)}$ |  | Sanches et al., 2014 |

**Supplementary Table 2.** Goodness-of-fit (R^2^) and accuracy (RMSE, MAE) for each case's top models, compared with the linear regression case; best and lowest RMSE are highlighted in bold font as pointed up in Figure 6.

|  |  |  | **All Bands** | | | **Selected Bands** | | | **VIs** | | |
| --- | --- | --- | --- | --- | --- | --- | --- | --- | --- | --- | --- |
| **Strategy** | **Time** | **Model** | **R^2^** | **RMSE** | **MAE** | **R^2^** | **RMSE** | **MAE** | **R^2^** | **RMSE** | **MAE** |
| **Sequential** |  | GPRSquaredExponential | 0.76 | 5.17 | 4.06 | 0.76 | **5.09** | 3.96 | 0.77 | **2.28** | 1.98 |
|  |  | RandomForest | 0.73 | 5.23 | 4.04 | 0.66 | 5.92 | 4.78 | 0.74 | 5.23 | 2.62 |
|  |  | MediumTree | 0.44 | 7.16 | 5.63 | 0.48 | **7.28** | 5.89 | 0.65 | 2.93 | 2.61 |
|  |  | SVRLinear | 0.75 | 5.21 | 4.17 | 0.75 | 5.18 | 4.14 | 0.76 | 3.77 | 2.47 |
|  |  | PLSR | 0.80 | **4.40** | 3.50 | 0.89 | 5.45 | 4.27 | 0.82 | 5.90 | 5.60 |
|  |  | Linear | 0.01 | **7.68** | 6.25 | 0.64 | 5.33 | 4.16 | 0.17 | **9.24** | 6.54 |
| **Retraining** | t1 | GPRSquaredExponential | 0.86 | **2.14** | 1.73 | 0.75 | **2.21** | 2.16 | 0.83 | 2.41 | 2.06 |
|  |  | RandomForest | 0.65 | 3.42 | 2.91 | 0.55 | 4.37 | 3.21 | 0.68 | 3.01 | 2.62 |
|  |  | MediumTree | 0.50 | 4.33 | 3.51 | 0.42 | 5.29 | 4.07 | 0.51 | 3.28 | 2.81 |
|  |  | SVRLinear | 0.68 | 3.24 | 2.64 | 0.61 | 3.92 | 3.26 | 0.67 | 3.77 | 2.97 |
|  |  | PLSR | 0.66 | 3.70 | 3.24 | 0.73 | 3.59 | 3.20 | 0.77 | **1.93** | 0.86 |
|  |  | Linear | 0.01 | **18.12** | 10.25 | 0.01 | **13.61** | 10.30 | 0.01 | **9.26** | 6.54 |
|  | t2 | GPRSquaredExponential | 0.59 | **2.71** | 2.13 | 0.69 | **4.08** | 3.37 | 0.74 | 4.80 | 3.80 |
|  |  | RandomForest | 0.24 | 4.44 | 3.73 | 0.20 | 6.11 | 4.58 | 0.60 | 4.55 | 3.29 |
|  |  | MediumTree | 0.10 | 6.00 | 4.70 | 0.09 | 7.65 | 6.24 | 0.44 | **6.04** | 4.90 |
|  |  | SVRLinear | 0.62 | 2.82 | 2.20 | 0.65 | 4.86 | 3.45 | 0.74 | 4.75 | 3.54 |
|  |  | PLSR | 0.08 | 5.43 | 4.08 | 0.95 | 4.84 | 3.49 | 0.61 | **2.56** | 0.98 |
|  |  | Linear | 0.02 | **11.37** | 8.93 | 0.01 | **21.24** | 17.25 | 0.01 | 5.54 | 3.86 |
|  | t3 | GPRSquaredExponential | 0.64 | **3.62** | 3.01 | 0.56 | 3.81 | 3.21 | 0.68 | 3.37 | 2.77 |
|  |  | RandomForest | 0.67 | 4.58 | 4.11 | 0.26 | 4.92 | 3.93 | 0.65 | 3.00 | 2.52 |
|  |  | MediumTree | 0.21 | 5.24 | 4.54 | 0.19 | 5.56 | 4.48 | 0.52 | 3.43 | 2.97 |
|  |  | SVRLinear | 0.61 | 3.94 | 2.80 | 0.54 | 4.35 | 3.57 | 0.66 | 5.80 | 3.61 |
|  |  | PLSR | 0.16 | 4.75 | 4.00 | 0.75 | **3.77** | 2.66 | 0.79 | **1.72** | 0.78 |
|  |  | Linear | 0.01 | 14.33 | 10.24 | 0.01 | **27.46** | 24.10 | 0.01 | **44.93** | 17.08 |
|  | t4 | GPRSquaredExponential | 0.61 | **4.49** | 3.99 | 0.48 | **4.02** | 3.07 | 0.64 | 4.03 | 3.37 |
|  |  | RandomForest | 0.42 | 7.62 | 6.22 | 0.28 | 5.89 | 4.52 | 0.56 | 3.88 | 3.12 |
|  |  | MediumTree | 0.26 | 8.52 | 7.20 | 0.13 | 6.44 | 4.82 | 0.41 | 4.83 | 3.91 |
|  |  | SVRLinear | 0.61 | 4.77 | 4.10 | 0.50 | 4.26 | 3.22 | 0.63 | 3.90 | 3.20 |
|  |  | PLSR | 0.56 | 5.15 | 4.54 | 0.97 | 6.37 | 5.30 | 0.44 | **3.35** | 1.53 |
|  |  | Linear | 0.01 | 21.74 | 17.23 | 0.08 | **25.32** | 13.56 | 0.01 | **17.06** | 11.51 |
|  | t5 | GPRSquaredExponential | 0.34 | 5.80 | 4.52 | 0.09 | 4.80 | 4.25 | 0.32 | 4.80 | 3.39 |
|  |  | RandomForest | 0.41 | **5.59** | 4.37 | 0.01 | 5.83 | 4.55 | 0.37 | 5.08 | 3.87 |
|  |  | MediumTree | 0.34 | 5.76 | 4.60 | 0.01 | 5.24 | 3.74 | 0.13 | 4.87 | 4.09 |
|  |  | SVRLinear | 0.15 | 6.05 | 4.96 | 0.01 | **4.07** | 3.26 | 0.03 | 5.14 | 3.87 |
|  |  | PLSR | 0.07 | 7.47 | 6.51 | 0.01 | 6.46 | 5.69 | 0.31 | **2.89** | 0.96 |
|  |  | Linear | 0.015 | **18.97** | 12.68 | 0.01 | **16.25** | 11.66 | 0.01 | **42.52** | 13.55 |

**Supplementary Table 3.** Relevant bands and VIs retrieved by the three selected models: random forest, GPR and PLSR. The fitted models under the sequential strategy were used for comparing the spectral bands, whereas their results under the retraining strategy were gathered for the VIs case. Consecutive bands are comprised in spectral ranges accordingly to Figure 9a.

| **Model** | **Relevant bands** | **Relevant VIs** | **Model** | **Relevant bands** | **Relevant VIs** | **Model** | **Relevant bands** | **Relevant VIs** |
| --- | --- | --- | --- | --- | --- | --- | --- | --- |
| Random  Forest | 400-410 | VREI1 | GPR | 405 | PRI | PLSR | 420-480 | SRI |
|  | 450 | VREI2 |  | 445-450 | CRI1 |  | 540-590 | LAI |
|  | 490 | REPI4 |  | 460-470 | CRI2 |  | 650-670 | MRESRI |
|  | 510-540 | SIPI |  | 530-540 | ARI2 |  | 690-710 | REPI4 |
|  | 550-570 | PSRI |  | 550-580 | WBI |  | 760-770 | CRI1 |
|  | 610 | CRI1 |  | 640-650 | SAVI |  | 800-830 | ARI1 |
|  | 620 | CRI2 |  | 655-660 | VREDI |  | 930-940 | WBI |
|  | 660 | MTVI2 |  | 695-710 | REPI1 |  | 980-1000 | MCARI |
|  | 690-710 | TCARI |  | 720-760 | REPI2 |  |  | MCARI/OSAVI |
|  | 770 | GNDVI |  | 765-770 | EGFN |  |  | NDCI2 |
|  | 790 | NDCI1 |  | 980-985 | Datt |  |  | Datt |
|  | 800 | D720 |  | 990-1000 | DPI |  |  | DSR2 |
|  | 890 | EGFN |  |  | FDNDVI |  |  | FDNDVI |
|  | 910-950 | Datt |  |  | AUC |  |  | ANMB |
|  | 960-1000 | DSR1 |  |  | ANMB |  |  | LPSDI |


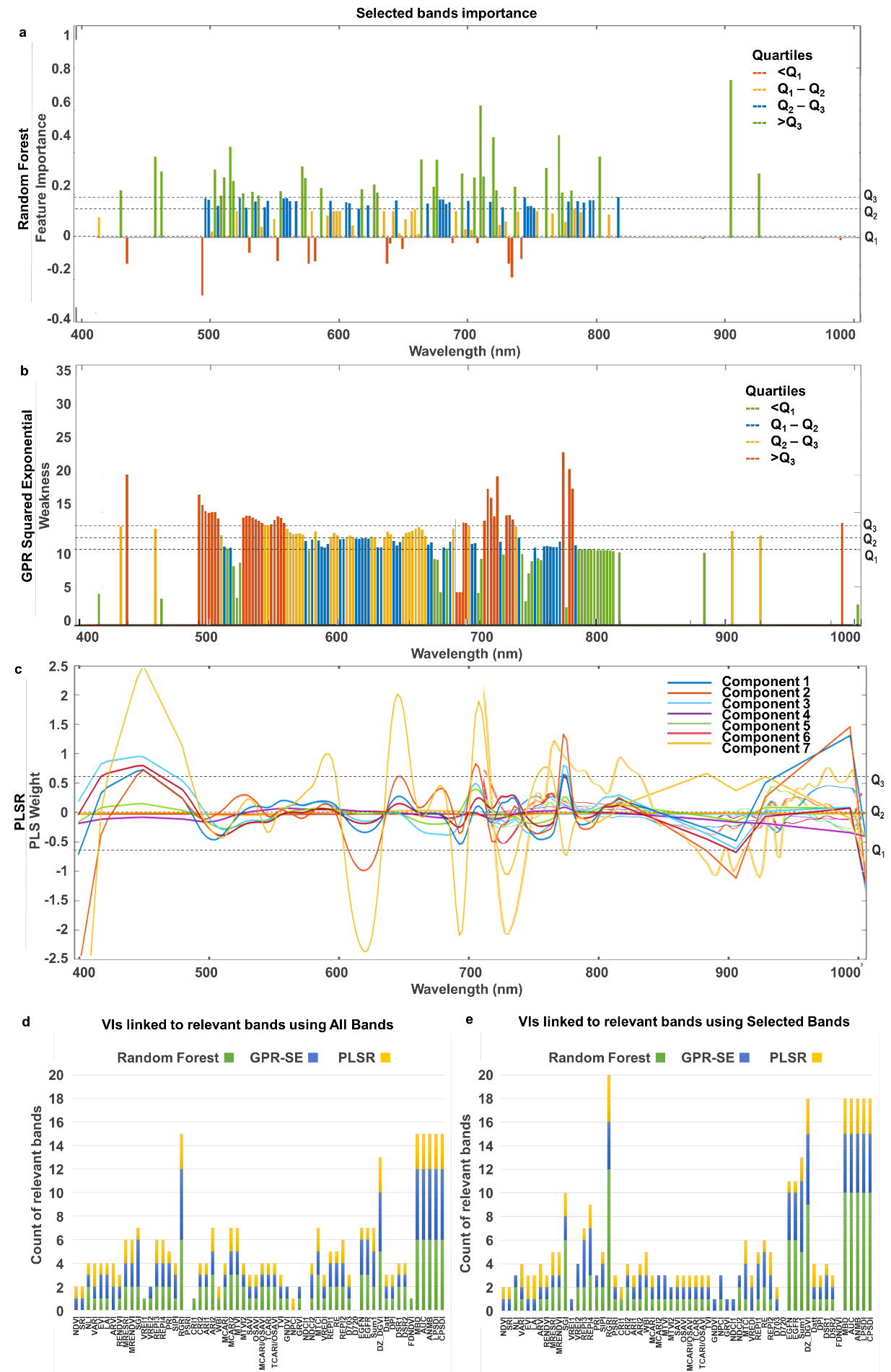


**Supplementary Figure 1.** Feature importance plots for the 145 bands subset. The quartile classification was used to rank the top features of each method. (a) For random forest, the third quartile threshold identifies the most important bands (Q3 = 0.19). (b) For GPR squared exponential, the first quartile classifies the less weak bands (Q1 = 10.01). (c) The predictor weights were retrieved for the first seven components used to fit the PLSR model, and the third quartile defines the most relevant bands (Q3 = 0.6). The relevant bands linked to one or more VIs were counted per VI and model under both band datasets: (d) using all bands, (e) using the selected 145 bands. For the all bands case (d), the relevant bands could be partially covered by most of the VIs, except for the PSRI, NDCI1, and D720, which registered zero bands.

**Supplementary Table 4.** Relevant bands from the 145 band subset retrieved by the three selected models: random forest, GPR and PLSR. The fitted models under the sequential strategy were used for comparing the two sets of spectral band predictors; the common relevant bands between them were marked with an asterisk. For the random forest case, several individual features (10 bands) and two spectral ranges (marked with an asterisk) coincide with the relevant features presented in Table 3. For the GPR squared exponential case, three spectral ranges (665-680 nm, 695-705 nm, 730-740 nm) and one individual band (970 nm) matched the features identified in Table 3. In contrast, for the PLSR model, only three spectral ranges (445-505 nm, 700-710 nm, 762-770 nm) were common between both band-based cases.

| **Model** | **Relevant bands** | | | | | | | | | | | |
| --- | --- | --- | --- | --- | --- | --- | --- | --- | --- | --- | --- | --- |
| Random Forest | 445* | 470 | 475 | 510* | 515-525* | 531* | 536* | 540* | 560* | 570* | 585 | 615* |
|  | 626-630 | 660* | 665 – 670 | 680 | 695-715* | 726 | 747 | 760-765 | 783 | 880 | 900 | 970* |
| GPR Squared Exp. | 430 | 475 | 520-530 | 585 | 665-680* | 695-705* | 715 | 730-740* | 762 | 770-800 | 860 | 970* |
| PLSR | 445-505* | 590-605 | 640-655 | 700-710* | 762-770* | 900 | 970 |  |  |  |  |  |
